# Supplementary material for: In vivo imaging of mitochondrial DNA mutations using an integrated nano Cas12a sensor
Source: Nat Commun. 2023 Nov 24;14:7722. doi: 10.1038/s41467-023-43552-0 (PMC10673915; doi:10.1038/s41467-023-43552-0)
Supplement: Supplementary file 1 — Supplementary Information [file 41467_2023_43552_MOESM1_ESM.pdf]

In Vivo Imaging of Mitochondrial DNA Mutations using an Integrated Nano Cas12a Sensor

Yanan Li[1], Yonghua Wu [1], Ru Xu[1], Jialing Guo[1], Fenglei Quan[1], Yongyuan Zhang[1], Di Huang[1], Yiran Pei[1], Hua Gao[1], Wei Liu[1], Junjie Liu[1], Zhenzhong Zhang[1,*], Ruijie Deng[2,*], Jinjin Shi[1,*], Kaixiang Zhang[1,*]

[1] School of Pharmaceutical Sciences, Key Laboratory of Targeting Therapy and Diagnosis for Critical Diseases, Collaborative Innovation Center of New Drug Research and Safety Evaluation, State Key Laboratory of Esophageal Cancer Prevention & Treatment, Zhengzhou University, Zhengzhou 450001, China

[2] College of Biomass Science and Engineering, Sichuan University, Chengdu 610065, China

*Corresponding authors' email addresses: zhangzhenzhong@zzu.edu.cn; drj17@scu.edu.cn; shijinyxy@zzu.edu.cn; zhangkx@zzu.edu.cn

Table of Contents

Supplementary Table 1. Oligonucleotides used in this study.

Supplementary Table 2. Fluorescent dyes used in this study.

Supplementary Figure 1. Preparation and characterization of InCasor.

Supplementary Figure 2. Subcellular localization of InCasor.

Supplementary Figure 3. Quantitative characterization of mitochondria targeting of InCasor.

Supplementary Figure 4. Long-term effects of InCasor on intracellular Mg^{2+} levels.

Supplementary Figure 5. *In vitro* analysis of $InCasor_{ND4}$ for targeted mtDNA detection.

Supplementary Figure 6. The self-degradation of InCasor inside the mitochondria.

Supplementary Figure 7. Gene editing efficiency and cytotoxicity of InCasor.

Supplementary Figure 8. Assessment of mtDNA specific site cleavage and off-target effects of mtDNA and nuclear DNA.

Supplementary Figure 9. Optimization of InCasor probe for live cell imaging.

Supplementary Figure 10. Preparation and characterization of the $InCasor_{ND4}$-Co^{2+} probe.

Supplementary Figure 11. Design InCasor for identifying mutations in mtDNA.

Supplementary Figure 12. Engineering crRNA for recognition of mutations in mtDNA that are distant from the PAM.

Supplementary Figure 13. Construction and validation of cells with heteroplasmic mtDNA.

Supplementary Figure 14. Imaging analysis of the heterogeneity of the 13105A>G mutation in the *ND5* gene in mtDNA in hybrid cells using $InCasor_{ND5\text{-}MT\text{-}1}$.

Supplementary Figure 15. Analysis of the *in vivo* biodistribution of InCasor.

Supplementary Figure 16. Analysis of the *in vivo* stability of InCasor.

Supplementary Figure 17. Analysis of *in vivo* safety of InCasor.

Supplementary Figure 18. Visualizing mtDNA mutation (12084C>T, SNV in PAM) *in vivo* using InCasor.

Supplementary Figure 19. Visualizing mtDNA mutation (13105A>G, SNV in near-PAM) *in vivo* using InCasor.

Supplementary Figure 20. Gating strategy for this work.

Supplementary Table 1. Oligonucleotides used in this study.

Name	Sequence (5'-3')
Template 1	P-TTGGTTCAGTTAGTTAATTTCGTAAATCAGTCATCTTTTGCAACAA CGTACCCAATGCGCCGGT
Template 2	P-CGGCCCCAGACACGGTTTTAACCGTACAGTATTTCCCGGCGGCGC AGCAGTTATT
Primer 1	AACTAACTGAACCAAAATAACTGCTGCGCC
Primer 2	CCGTGTCTGGGGCCGACCGGCGCATTGGGT
Template 2-no Sgc 8	P-CGGCCCCAGACACGGTTTTTTTTTTTTTTTTTTTTTTTTTTTTTTTTT TTTTTTTT
Primer1 no Sgc8	AACTAACTGAACCAAAAAAAAAAAAAAAAA
Template1-no Cyt C apt	P-TTGGTTCAGTTAGTTAATTTCGTAAATCAGTCATCTTTTTTTTTTTT TTTTTTTTTTTTTTT
Template 2-no Cyt C apt	P-TTTTTTTTTTTTTTTTTTTTAACCGTACAGTATTTCCCGGCGGCGCA GCAGTTATT
Primer 2-no Cyt C apt	AAAAAAAAAAAAAAAAAAAAAAAAAAAAAA
FAM-reporter	T(FAM)TATT(BHQ-1)
ssDNA-FAM-reporter	P-TGATT(FAM)TATT(BHQ-1)ACGTAAATCAGTCATCGAGAC
ssDNA-Cy3-repoter	P-TGATT(Cy 3)TATT(BHQ-2)ACGTAAATCAGTCATCGAGAC
ssDNA-Cy5-reporter	P-TGATT(Cy 5)TATT(BHQ-2)ACGTAAATCAGTCATCGAGAC
NTS_{ND4}	AACAACAACCTA**TTTA**GCTGTTCCCCAACCTTTTCCTCCGACCCCCTA ACAACCCCCC
TS_{ND4}	GGGGGGTTGTTAGGGGGTCGGAGGAAAAGGTTGGGGAACAGC**TAAA** TAGGTTGTTGTT
$crRNA_{ND4}$	GGUUCAGUUAGUUAAUUUUUUAAUUUCUACUAAGUGUAGAUGCUG UUCCCCAACCUUUUCC
crRNA F-Primer	TAATACGACTCACTATAGGG
Template $crRNA_{ND4}$	TAATACGACTCACTATAGGGTTCAGTTAGTTAATTTTTTAATTTCTACT AAGTGTAGATGCTGTTCCCCAACCTTTTCC
R-Primer $_{ND4}$	GGAAAAGGTTGGGGAACAGC
NTS_{ND4-2}-WT	CATACACCTATTCCCCA**TCC**TCCTCCTCCTATCCCTCAACCCCGACATC ATTA
TS_{ND4-2}-WT	TAATGATGTCGGGGTTGAGGGATAGGAGGAGGAGAATGGG**GGA**TAG GTGTATG

NTS_{ND4-2}-MT	CATACACCTATTCCCCATTCTCCTCCTCCTATCCCTCAACCCCGACATCATTA
TS_{ND4-2}-MT	TAATGATGTCGGGGTTGAGGGATAGGAGGAGGAGAATGGGGAATAGGTGTATG
$crRNA_{ND4-2}$	GGUUCAGUUAGUUAAUUUUUUAAUUUCUACUAAGUGUAGAUUCCUCCUCCUAUCCCUC
Template $crRNA_{ND4-2}$	TAATACGACTCACTATAGGGTTCAGTTAGTTAATTTTTTAATTTCTACTAAGTGTAGATCCCATTCTCCTCCTCCTATC
R-$Primer_{ND4-2}$	GATAGGAGGAGGAGAATGGG
NTS_{ND5} -WT	CCACTCAAGCACTATAGTTGTAGCAGGAATCTTCTTACTCATCCGCTTCCAC
TS_{ND5} -WT	GTGGAAGCGGATGAGTAAGAAGATTCCTGCTACAACTATAGTGCTTGAGTGG
NTS_{ND5} -MT	CCACTCAAGCACTATAGTTGTAGCAGGAGTCTTCTTACTCATCCGCTTCCAC
TS_{ND5}-MT	GTGGAAGCGGATGAGTAAGAAGACTCCTGCTACAACTATAGTGCTTGAGTGG
$crRNA_{ND5}$	GGUUCAGUUAGUUAAUUUUUUAAUUUCUACUAAGUGUAGAUUAGCAGGAGUCUUCUUACUC
Template $crRNA_{ND5}$	TAATACGACTCACTATAGGGTTCAGTTAGTTAATTTTTTAATTTCTACTAAGTGTAGATTAGCAGGAGTCTTCTTACTC
R-$Primer_{ND5}$	GAGTAAGAAGACTCCTGCTA
1-bp mismatch crRNA	GGUUCAGUUAGUUAAUUUUUUAAUUUCUACUAAGUGUAGAUUAGCAGGAGACUUCUUACUC
Template 1-bp mismatch crRNA	TAATACGACTCACTATAGGGTTCAGTTAGTTAATTTTTTAATTTCTACTAAGTGTAGATTAGCAGGAGACTTCTTACTC
R-Primer 1-bp mismatch	GAGTAAGAAGTCTCCTGCTA
2-bp mismatch crRNA	GGUUCAGUUAGUUAAUUUUUUAAUUUCUACUAAGUGUAGAUUAGCAGGAGAGUUCUUACUC
Template 2-bp mismatch crRNA	TAATACGACTCACTATAGGGTTCAGTTAGTTAATTTTTTAATTTCTACTAAGTGTAGATTAGCAGGAGAGTTCT TAC TC
R-Primer 2-bp mismatch	GAGTAAGAACTCTCCTGCTA
3-bp mismatch crRNA	GGUUCAGUUAGUUAAUUUUUUAAUUUCUACUAAGUGUAGAUUAGCAGGAGAGAUCUUACUC

Template 3-bp mismatch crRNA	TAATACGACTCACTATAGGGTTCAGTTAGTTAATTTTTTAATTTCTACTAAGTGTAGATTAGCAGGAGAGATCTTACTC
R-Primer 3-bp mismatch	GAGTAAGATCTCTCCTGCTA
4-bp mismatch crRNA	GGUUCAGUUAGUUAAUUUUUUAAUUUCUACUAAGUGUAGAUUAGCAGGAGAGAACUUACUC
Template 4-bp mismatch crRNA	TAATACGACTCACTATAGGGTTCAGTTAGTTAATTTTTTAATTTCTACTAAGTGTAGATTAGCAGGAGAGAACTTACTC
R-Primer 4-bp mismatch	GAGTAAGTTCTCTCCTGCTA
Cytochrome C aptamer	CGATTTACACCCGGTGATTTTCCGTGTCTGGGGCCGACCGGCGCATTGGGTACGTTGTTGC
$crRNA_{NT}$	GGUUCAGUUAGUUAAUUUUUUAAUUUCUACUAAGUGUAGAUUGUCGUCCGAAAUACUACCG
Template $crRNA_{NT}$	TAATACGACTCACTATAGGGTTCAGTTAGTTAATTTTTTAATTTCTACTAAGTGTAGATTGTCGTCCGAAATACTACCG
R-Primer $crRNA_{NT}$	CGGTAGTATTTCGGACGACA
F-$Primer_{ND4}$	AACATTAACGAAAATAACCCC
R-$Primer_{ND4}$	TTTTTGGGTTGAGGTGATGA
F-$Primer_{ND4-2}$	ATCATAATCCTCTCTCAAGG
R-$Primer_{ND4-2}$	AAGGGGTCGTAAGCCTCTGT
5'-$crRNA_{ND4}$	CGT AAA TCA GTC ACT<br>UUUUUUUUUUUUUUU<br>GGUUCAGUUAGUUAAUUUUUUAAUUUCUACUAAGUGUAGAUGCUGUUCCCCAACCUUUUCC
Template 5'-$crRNA_{ND4}$	TAATACGACTCACTATAG CGTAAATCAGTCACT TTTTTTTTTTTTTTT GGTTCAGTTAGTTAATTTTTTAATTTCTACTAAGTGTAGATGCTGTTCCCCAACCTTTTCC
5'-$crRNA_{ND4}$-FAM	AGTGACTGATTTACG-FAM
Au-DNF	SH-TTTTTTTTTTTTTTTTTTTT CGT AAA TCA GTC ATC
Au-crRNA	A GTG ACT GAT TTA CG TTTTTTTTTTTTTTTTTTTT-SH
Template-crRNA	AACTGAACCAAAACTCAATTCTGCTTGACAACTACTGCGTCTATTTTCACCTC GCA TA AAA AAA TTA ACT
Cas13a-crRNA	GAUUUAGACUACCCCAAAAACGAAGGGGACUAAAACAT CTA CAC TTA GTA GAA ATT
Template Cas13a-crRNA	TAA TAC GAC TCA CTA TAGGG GATTTAGACTACCCCAAAAACGAAGGGGACTAAAACATCTACACTTAGTAGAAATT

R-Primer Cas13a-crRNA	AATTTCTACTAAGTGTAGAT
Poly U Reporter	U(FAM)UUUU(BHQ1)
PS-F-Primer	CAGCCATAGAAGGCCCCA
PS-R-Primer	Bio-GTTTGGATTAGTGGGCTATTTTC
PS-PS-Primer	TCAAGCACTATAGTTGTAGCAGG
Padlock-WT	P-TCCTGCTACAACTATTCCTTTTACGACCTCAATGCTGCTGCTGTACTACTCTTCGATGAGTAAGAAGAT
Imager-WT	C(Cy3)CTCAATGCTGCTGCTGTACTAC
Padlock-MT	P-TCCTGCTACAACTACCTTTCCTACGACCTCAATGCACATGTTTGGCTCCTCTTCGATGAGTAAGAAGAC
Imager-MT	C(AF488)CTCAATGCACATGTTTGGCTCC
Template $_{MUC-1}$	P-TTGGTTCAGTTAGTTAATTTTCGTAAATCAGTCATCTTTTGCAACAACGTACCCAATGCGCCGGTCGGCCCCAGACACGGTTTTGTCGTTTTACCCATGTGCTATAGCCTT
Primer $_{MUC-1}$	AACTAACTGAACCAAAAGGCATAGCACAT
NTS$_{ND2}$-WT	ATACTCATCA**TTA**ATAATCATAATAGCTATAGCAATAAAACTA
TS$_{ND2}$-WT	TAGTTTTATTCGTATAGCTATTATGATTAT**TAA**TGATGAGTAT
NTS$_{ND2}$-MT	ATACTCATCA**TTA**ATAATCATAAT**G**GCTATAGCAATAAAACTA
TS$_{ND2}$-MT	TAGTTTTATTCGTATAGC**C**ATTATGATTAT**TAA**TGATGAGTAT
crRNA$_{ND2-FM}$	GGUUCAGUUAGUUAAUUUUUUAAUUUCUACUAAGUGUAGAUAUAAU CAUAA U**G**GCU AUAGC
Template crRNA$_{ND2-FM}$	TAATACGACTCACTATAGGGTTCAGTTAGTTAATTTTTTAATTTCTACTAAGTGTAGATATAATCATAATGGCTATAGC
R-Primer$_{ND2-FM}$	GCTATAGCCATTATGATTAT
crRNA$_{ND2-SM1}$	GGUUCAGUUAGUUAAUUUUUUAAUUUCUACUAAGUGUAGAUAU**U**AU CAUAA U**G**GCU AUAGC
Template crRNA$_{ND2-SM1}$	TAATACGACTCACTATAGGGTTCAGTTAGTTAATTTTTTAATTTCTACTAAGTGTAGATATTATCATAATGGCTATAGC
R-Primer$_{ND2-SM1}$	GCTATAGCCATTATGATAAT
crRNA$_{ND2-SM2}$	GGUUCAGUUAGUUAAUUUUUUAAUUUCUACUAAGUGUAGAUAUAAU CAUAA**AG**GCU AUAGC
Template crRNA$_{ND2-SM2}$	TAATACGACTCACTATAGGGTTCAGTTAGTTAATTTTTTAATTTCTACTAAGTGTAGATATAATCATAAAGGCTATAGC

R-Primer$_{ND2-SM2}$	GCTATAGCCTTTATGATTAT
crRNA$_{ND2-DM1}$	GGUUCAGUUAGUUAAUUUUUUAAUUUCUACUAAGUGUAGAUAUUA A CAUAAUGGCU AUAGC
Template crRNA$_{ND2-DM1}$	TAATACGACTCACTATAGGGTTCAGTTAGTTAATTTTTTAATTTCTACT AAGTGTAGATATTAACATAATGGCTATAGC
R-Primer$_{ND2-DM1}$	GCTATAGCCATTATGTTAAT
crRNA$_{ND2-DM2}$	GGUUCAGUUAGUUAAUUUUUUAAUUUCUACUAAGUGUAGAUAUAA U CAUAUAGGCU AUAGC
Template crRNA$_{ND2-DM2}$	TAATACGACTCACTATAGGGTTCAGTTAGTTAATTTTTTAATTTCTACT AAGTGTAGATATAATCATATAGGCTATAGC
R-Primer$_{ND2-DM2}$	GCTATAGCCTATATGATTAT
NTS$_{ND1}$-WT	CCGAACCCCCTTCGACCTTGCCGAAGGGGAGTCCGAACTAGTC
TS$_{ND1}$-WT	GACTAGTTCGGACTCCCCTTCGGCAAGGTCGAAGGGGGTTCGG
NTS$_{ND1}$MT	CCGAACCCCCTTCGACCTTGCCGAAGGGAAGTCCGAACTAGTC
TS$_{ND1}$-MT	GACTAGTTCGGACTTCCCTTCGGCAAGGTCGAAGGGGGTTCGG
crRNA$_{ND1-FM}$	GGUUCAGUUAGUUAAUUUUUUAAUUUCUACUAAGUGUAGAUGACC U UGCCGAAGGGAAGUC
Template crRNA$_{ND1-FM}$	TAATACGACTCACTATAGGGTTCAGTTAGTTAATTTTTTAATTTCTACT AAGTGTAGATGACCTTGCCGAAGGGAAGTC
R-Primer$_{ND1-FM}$	GACTTCCCTTCGGCAAGGTC
crRNA$_{ND1-SM1}$	GGUUCAGUUAGUUAAUUUUUUAAUUUCUACUAAGUGUAGAUGAGC UUGCCGAAGGGAAGUC
Template crRNA$_{ND1-SM1}$	TAATACGACTCACTATAGGGTTCAGTTAGTTAATTTTTTAATTTCTACT AAGTGTAGATGAGCTTGCCGAAGGGAAGTC
R-Primer$_{ND1-SM1}$	GACTTCCCTTCGGCAAGCTC
crRNA$_{ND1-SM2}$	GGUUCAGUUAGUUAAUUUUUUAAUUUCUACUAAGUGUAGAUGACC UUGCCGAAGGCAAGUC
Template crRNA$_{ND1-SM2}$	TAATACGACTCACTATAGGGTTCAGTTAGTTAATTTTTTAATTTCTACT AAGTGTAGATGACCTTGCCGAAGGCAAGTC
R-Primer$_{ND1-SM2}$	GACTTGCCTTCGGCAAGGTC
crRNA$_{ND1-DM1}$	GGUUCAGUUAGUUAAUUUUUUAAUUUCUACUAAGUGUAGAUGAGC AUGCCGAAGGGAAGUC

Template crRNA$_{ND1-DM1}$	TAATACGACTCACTATAGGGTTCAGTTAGTTAATTTTTTAATTTCTACTAAGTGTAGATGAGCATGCCGAAGGGAAGTC
R-Primer$_{ND1-DM1}$	GACTTCCCTTCGGCATGCTC
crRNA$_{ND1-DM2}$	GGUUCAGUUAGUUAAUUUUUUAAUUUCUACUAAGUGUAGAUGACCU UGCCGAAG**CCA**AGUC
Template crRNA$_{ND1-DM2}$	TAATACGACTCACTATAGGGTTCAGTTAGTTAATTTTTTAATTTCTACTAAGTGTAGATGACCTTGCCGAAGCCAAGTC
R-Primer$_{ND1-DM2}$	GACTTGGCTTCGGCAAGGTC

PAM sequence of Cas12a is indicated by bold orange letter. Targe site in dsDNA of Cas12a is indicated by underscore. SNV is indicated by bold red letter. Each mismatched position is indicated by bold blue letter.

Supplementary Table 2. Fluorescent dyes used in this study.

Name	Ex (nm)	Em (nm)	Manufacturer
Hoechst	405	430-460	Beyotime
FAM	488	500-540	Sangon Biotech
Mito Tracker Red	552	570-600	Beyotime
Mito Tracker Green	488	500-540	Beyotime
Cy 3	552	570-600	Sangon Biotech
Cy 5	638	650-670	Sangon Biotech
DiI	552	570-600	Beyotime
DiD	638	650-670	Beyotime
Cell Mask Green	488	500-540	Thermo Fisher
DAPI	405	430-460	Beyotime
FITC	488	500-540	Abcam

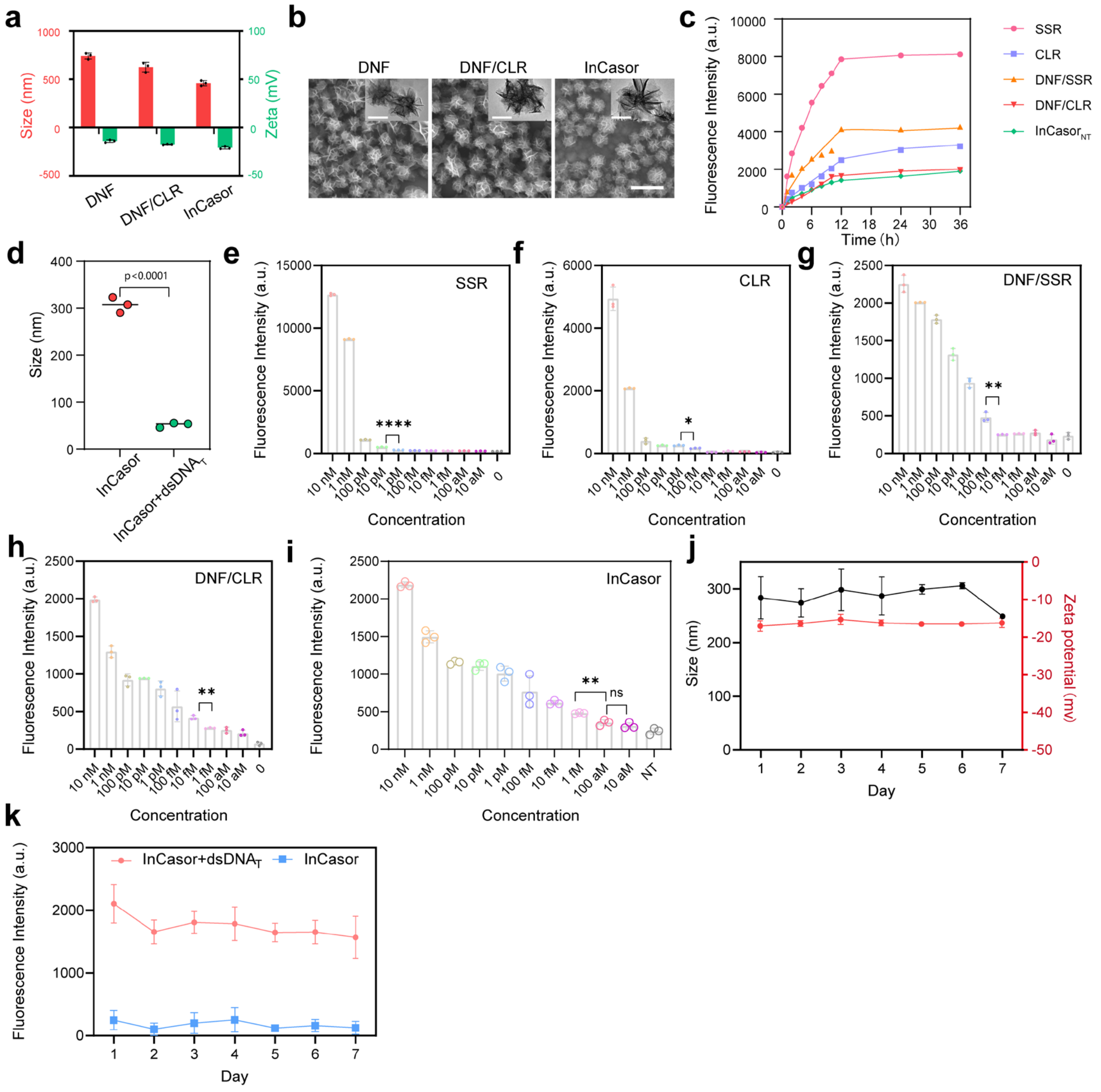

Supplementary Fig. 1. Preparation and characterization of InCasor. a. Particle sizes and Zeta potential of DNF, DNF/CLR and InCasor probe. (n = 3). Data are presented as mean ± SD. **b.** SEM characterization of DNF, DNF/CLR and InCasor probe. Scale bar: 1 μm. Inset shows the corresponding TEM image. Scale bar: 200 nm. The experiments were repeated three times independently. **c.** *In vitro* fluorescence assay to analyze the stability of InCasor in 90% serum over time. n=6 biologically independent experiment, data show mean ± SD. **d.** DLS analysis of particle size changes of InCasor before and after recognition of target mtDNA *in vitro*. n=3 biologically independent experiment, data are analyzed by two-sided Student's t-test and shown as mean ± SD. **e-i.** Comparison of the mtDNA detection efficiency of the SSR (e), CLR (f), DNF/SSR (g), DNF/CLR (h), and InCasor (i). n=3 biologically independent experiment, data show mean ± SD. **j.** Variation of particle size and potential of InCasor with time at 4 °C storage conditions. n=3 biologically independent experiment, data show mean ± SD. **k.** The ability of InCasor to recognize target DNA to produce a fluorescent signal over time under storage conditions at 4 °C. n=3 biologically independent experiment, data show mean ± SD. Source data from (a, and c-k) are

provided as a Source Data file.

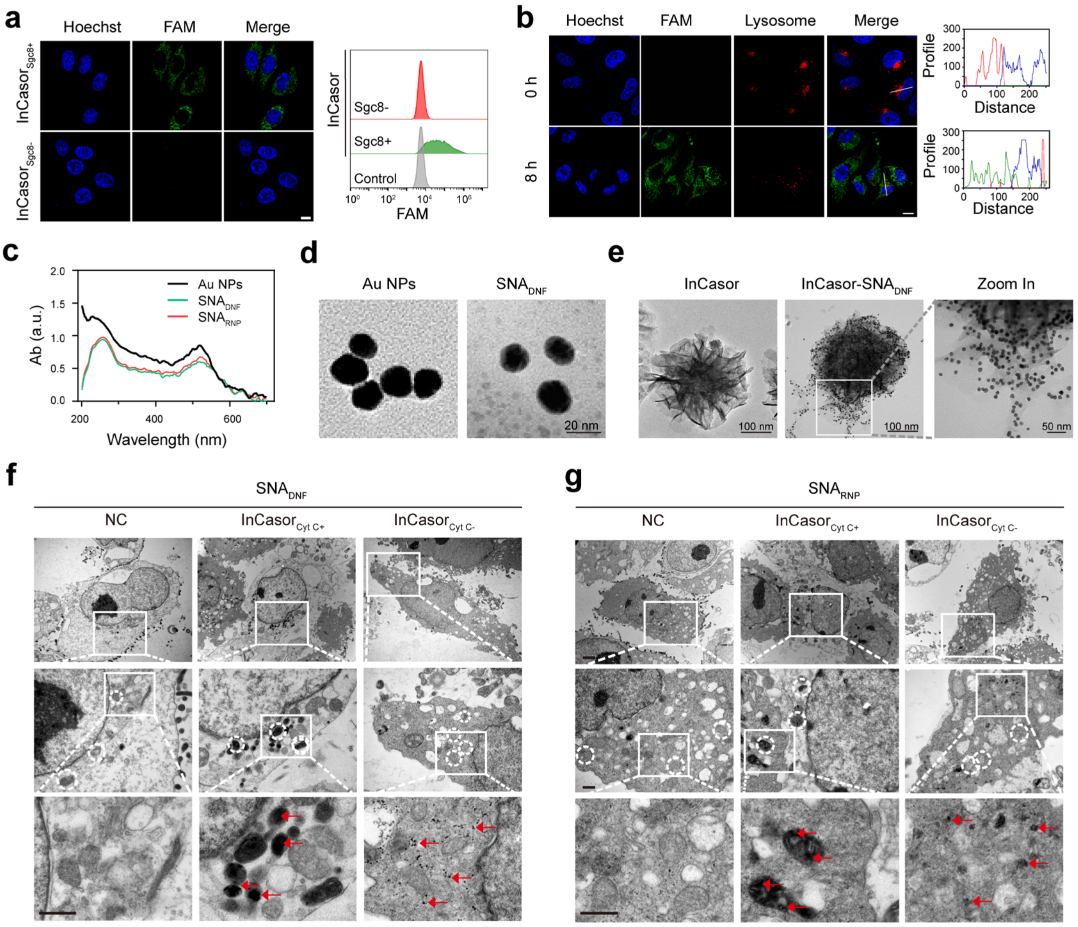

Supplementary Fig. 2. Subcellular localization of InCasor. a. Confocal imaging and flow cytometry analysis of HepG2 cells incubated with 50 nM FAM-InCasor$_{Sgc8+}$ or FAM-InCasor$_{Sgc8-}$. Scale bar: 10 μm. **b.** Fluorescent imaging of the subcellular localization of InCasor in HepG2 cells after different incubation time. InCasor was labelled with FAM (green), and lysosomes were stained with Lysotracker (red). Scale bar: 10 μm. The spatial co-localization of InCasor and lysosomes were statically analyzed by Fiji. **c**. UV absorption spectrum of Au NPs, SNA$_{DNF}$ and SNA$_{RNP}$. **d.** TEM characterization of Au NPs and SNA$_{DNF}$. Scale bar: 20 nm. **e.** TEM characterization of InCasor and InCasor- SNA$_{DNF}$. **f.** Bio-TEM of HepG2 cells treated with SNA$_{DNF}$ labelled InCasor$_{Cyt\ C\ apt+}$ or InCasor$_{Cyt\ C\ apt-}$. **g.** Bio-TEM of HepG2 cells treated with SNA$_{RNP}$ labelled InCasor$_{Cyt\ C\ apt+}$ or InCasor$_{Cyt\ C\ apt-}$. Mitochondria are circled with white dotted lines. The red arrow points to SNA. The scale bars are 2 μm, 500 nm and 500 nm, respectively. The experiments were repeated three times independently. Source data from (b, c) are provided as a Source Data file.

Notes to Supplementary Fig. 2 c-g.

We performed Bio-TEM experiments for HepG2 cells treated with InCasor$_{Cyt\ C\ apt+}$ or InCasor$_{Cyt\ C\ apt-}$. First of all, different spherical nucleic acids (SNA) were synthesized

for labeling DNF and Cas12a RNP on InCasor respectively) for Bio-TEM experiments. In brief, we designed a two-domain DNA comprising an DNF domain (for labeling DNF, the DNA domain is similar to apart of CLR; for labeling Cas12a RNP, the DNA domain is complementary to 5` of crRNA, blue lines) and SH-modified poly T (T1 to T20) domain (red lines, sequences listed in Table S1). The SH-modified poly T -DNAs were assembled with AuNPs (~15 nm diameter) via a freezing method as described[1]. UV absorption spectrum of the DNA-AuNP conjugates show characteristic absorption peaks of nucleic(λ_{260nm}) acid and AuNP(λ_{520nm}) at the same time (**Supplementary Fig. 2c**) and transmission electron microscopy (TEM) images of the DNA-AuNP conjugates show a thin and low-contrast shell coating on the AuNPs (**Supplementary Fig. 2d**), suggesting that the polyT-DNAs were successfully attached to AuNPs. From the TEM of InCasor-SNA_{DNF}, the SNA_{DNF} were successfully labelled on InCasor probe (**Supplementary Fig. 2e**). Then, Bio-TEM was performed to show the location of $InCasor_{Cyt\ C\ apt+}$ or $InCasor_{Cyt\ C\ apt-}$ labelled by SNA_{DNF} or SNA_{RNP} in the HepG2 cells. As you can see from the following **Supplementary Fig. 2 f-g**, numerous SNA labelled $InCasor_{Cyt\ C\ apt+}$ probe was located mitochondria and part of it was in the endosomes/ lysosomes, cytoplasm. Meanwhile, there are no Au NPs in the mitochondria of untreated cells or SNA labelled $InCasor_{Cyt\ C\ apt-}$ treated cells. These results reveal that InCasor probe effectively targets mitochondria in living HepG2 cells.

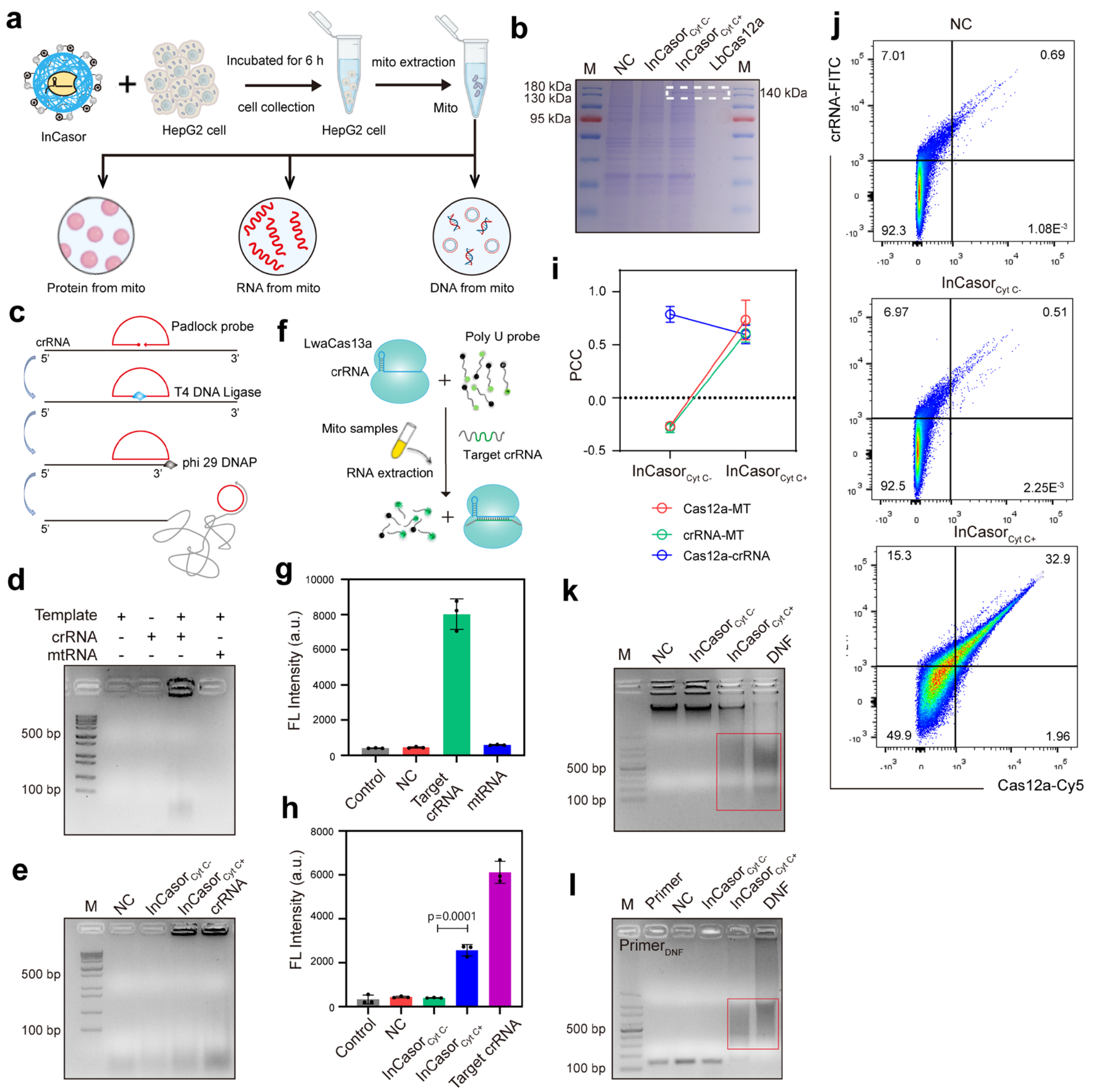

Supplementary Fig. 3. Quantitative characterization of mitochondria targeting of InCasor. a. Workflow for quantitative characterization of mitochondria targeted by InCasor. First, InCasor$_{Cyt\,C\,apt+}$ or InCasor$_{Cyt\,C\,apt-}$ probe were incubated with HepG2 cells for 6 h. After that, the cells were collected and the mitochondria in them were isolated. Proteinase, RNase, and DNase protection assays were used to degrade InCasor adsorbed on the outer mitochondrial membrane, beforehand extracting DNA, protein or RNA from the mitochondria for the next step of quantitative analysis. **b.** Analysis of proteins in Mito extracted from HepG2 cells after incubated with InCasor$_{Cyt\,C\,apt+}$ or InCasor$_{Cyt\,C\,apt-}$ probe through SDS-PAGE gel. The proteins were stained by coomassie brilliant blue. **c.** Schematic diagram of RCA method for specific crRNA detection. **d.** Feasibility analysis of crRNA triggering RCA reaction. **e.** Agarose gel electrophoresis analysis of the product of the RCA reaction initiated by crRNA in Mito extracted from HepG2 cells after incubated with InCasor$_{Cyt\,C\,apt+}$ or InCasor$_{Cyt\,C\,apt-}$ probe. **f.** Schematic diagram of Cas13a method for specific crRNA detection. **g.** In the presence of target crRNA, the trans-cleavage activity of Cas13a is able to in to generate fluorescence signal. n=3, data show mean ± SD. **h.** Fluorescence analysis of the Cas13a RNP incubated with RNA in Mito extracted from HepG2 cells after incubated with

InCasor$_{Cyt\ C\ apt+}$ or InCasor$_{Cyt\ C\ apt-}$ probe. n=3, data are analyzed by two-sided Student's t-test and shown as mean ± SD. **i.** The Pearson's correlation coefficient (PCC) of Cas12a RNP on InCasor and mitochondrial in Fig. 2e were statically analyzed by Fiji. n=3, data show mean ± SD. **j.** Quantification of Cy5-Cas12a/ FITC-crRNA in mitochondria using flow cytometry. HepG2 cells were incubated with InCasor$_{Cyt\ C\ apt+}$ or InCasor$_{Cyt\ C\ apt-}$ probe (both loading Cy5-Cas12a/ FITC-crRNA). Cells without any transfection were used as the negative control (NC). Crude mitochondria were isolated from cells at 12 h after transfection, and were subjected to flow cytometry. **k.** Agarose gel electrophoresis analysis of DNA in Mito extracted from HepG2 cells after incubated with InCasor$_{Cyt\ C\ apt+}$ or InCasor$_{Cyt\ C\ apt-}$ probe. **l.** Agarose gel electrophoresis analysis of PCR product of DNA in Mito extracted from HepG2 cells after incubated with InCasor$_{Cyt\ C\ apt+}$ or InCasor$_{Cyt\ C\ apt-}$ probe. The experiments were repeated three times independently. Source data from (b-e, g-I, and k-l) are provided as a Source Data file.

Notes to Supplementary Fig. 3 d-l.

A padlock probe based rolling circle amplification (RCA) methods was firstly applied based on the principles described in **Supplementary Fig. 3c** [2,3]. Padlock probes hybridize to their target sequence (crRNA) and the probe ends are joined through ligation, locking the probe onto the target molecule. After ligation, the RCA is initiated by the Phi 29 DNA polymerase, which turns the target molecule into a primer by 3`-5` exonucleolytic cleavage of any 3`end protruding from the hybridization site of the padlock probe. The padlock probe then serves as the template for DNA synthesis. The RCA product (gray) is detected through agarose gel electrophoresis. After verifying the feasibility of this method (**Supplementary Fig. 3d**), we examined whether total RNA of mitochondria in HepG2 cells with different treatments can triggered RCA responses. Like the positive control crRNA group, total RNA in InCasor$_{Cyt\ C\ apt+}$ group was also able to elicit RCA responses, while neither InCasor$_{Cyt\ C\ apt-}$ group nor untreated group showed RCA products (**Supplementary Fig. 3e**). These results suggested that there was crRNA of InCasor probe in the total RNA sample extracted from mito of InCasor$_{Cyt\ C\ apt+}$ group.

Moreover, a CRISPR-Cas13a based fluorescence assay was employed to detect the crRNA in total RNA sample. A CRISPR guide RNA (crRNA) complementary to 20 nucleotides of the crRNA of Cas12a was used to direct Cas13 from Leptotrichia wadei (LwaCas13a) to the target sequence. Detection of the target resulted in Cas13 activation and subsequent collateral cleavage of an oligonucleotide carrying a quenched

fluorophore that exhibits fluorescence when cleaved, correlating with the initial concentration of the crRNA in the total RNA sample from mito (**Supplementary Fig. 3f**). As shown in **Supplementary Fig. 3g**, only the target crRNA can trigger the trans-cleavage activity of Cas13a, resulting in an obvious fluorescent signal. We, therefore, continued to use this method to detect the presence of crRNA in different RNA samples. The significantly increased fluorescence signal in $\text{InCasor}_{\text{Cyt C apt+}}$ group demonstrated the presence of crRNA (**Supplementary Fig. 3h**). Together, the results indicated that our InCasor probe can delivery crRNA to mitochondria in living HepG2 cells.

In addition, we analyzed the DNA (equal mass) from mito in HepG2 cells by agarose gel electrophoresis (**Supplementary Fig. 3k**). The $\text{InCasor}_{\text{Cyt C apt+}}$ group had diffuse bands around 500 bp, which were similar to the DNF positive control group after acid treatment[4], indicating that these fragments may be produced by InCasor probe in mitochondria. To further verify that these bands were derived from InCasor probe, we performed PCR with DNA extracted from mitochondria using DNF-specific primers. As shown in **Supplementary Fig. 3l**, the diffuse band circled by the red box indicated that the DNF of InCasor probe was in the mitochondria.

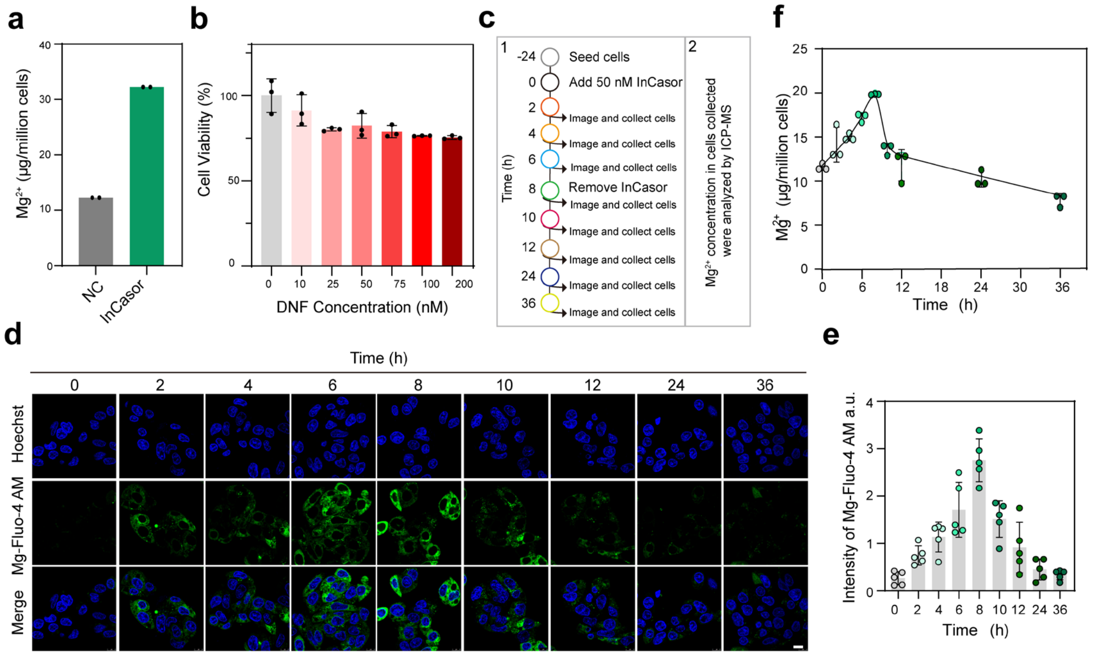

Supplementary Fig. 4. Long-term effects of InCasor on intracellular Mg^{2+} levels. **a.** ICP-MS analysis of Mg^{2+} concentration in HepG2 cells treated with 50 nM InCasor. n=2, data show mean ± SD. **b.** Cell viability analysis of HepG2 cells treated with different concentrations of DNF. n=3, data show mean ± SD. **c.** Schematic for analysis the intracellular Mg^{2+} levels in HepG2 cells treated with 50 nM InCasor in a long time. **d.** Confocal imaging was used to analyze the fluorescence signal of Mg-Fluo-4 AM in HepG2 cells treated by 50 nM InCasor for different times. **e.** Semi-quantitative statistics of Mg-Fluo-4 AM in HepG2 cells after different treatments. Data are expressed as mean ± SD (n = 5, data show mean ± SD.). **f.** The result of using ICP-MS to analyze Mg^{2+} concentration in HepG2 cells treated with 50 nM InCasor for different times (n = 3, data show mean ± SD.). Source data from (a-b, f, and e) are provided as a Source Data file.

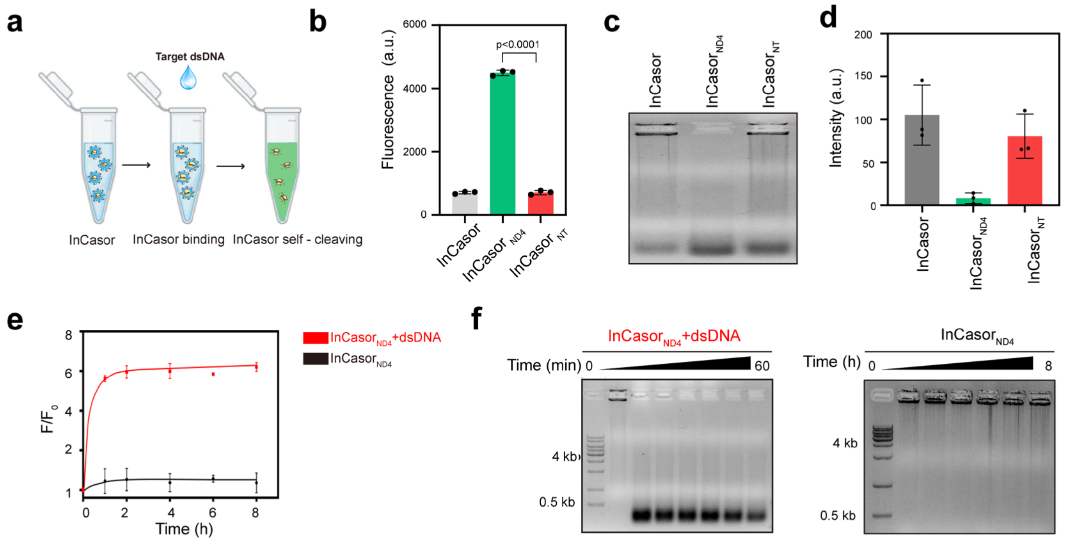

Supplementary Fig. 5. *In vitro* analysis of InCasor$_{ND4}$ for targeted mtDNA detection. a. Schematics of *in vitro* self-cleavage assay of InCasor$_{ND4}$, activated by *ND4* gene in mtDNA. **b.** In the presence of target mtDNA, InCasor$_{ND4}$ is able to generate fluorescence signal. n=3, data show mean ± SD. **c-d.** Agarose gel electrophoresis analysis and its quantitative analysis of self-degradation of InCasor$_{ND4}$ after incubated with target mtDNA. n=3, data show mean ± SD. **e.** Representative time-fluorescence curve of InCasor$_{ND4}$ incubated with target mtDNA. n=3, data show mean ± SD. **f.** Agarose gel electrophoresis analysis of InCasor$_{ND4}$ incubated with/without target mtDNA. The experiments were repeated three times independently. Source data from (b-f) are provided as a Source Data file.

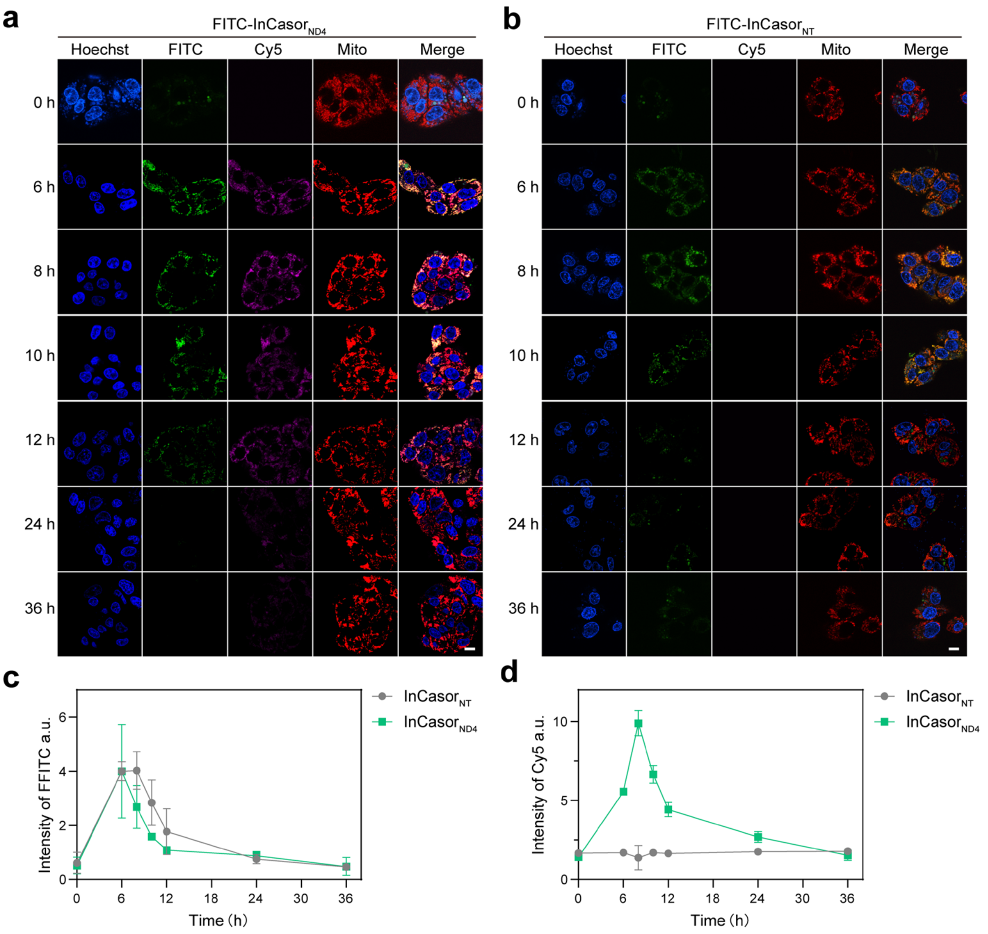

Supplementary Fig. 6. The self-degradation of InCasor inside the mitochondria. a-b. Confocal imaging was used to analyze the intensity of FITC fluorescence signal and Cy5 fluorescence signal in HepG2 cells changed with time after FITC-InCasor$_{ND4}$ (a) or FITC-InCasor$_{NT}$ (b) treatment. Scale bar: 10 μm. **c.** Semi-quantitative analysis of fluorescent intensity of FITC in panel a-b is shown. n=5 biological repeats. **d.** Semi-quantitative analysis of fluorescent intensity of Cy5 in panel a-b is shown. n=5 biological repeats, data show mean ± SD. Source data from (c and d) are provided as a Source Data file.

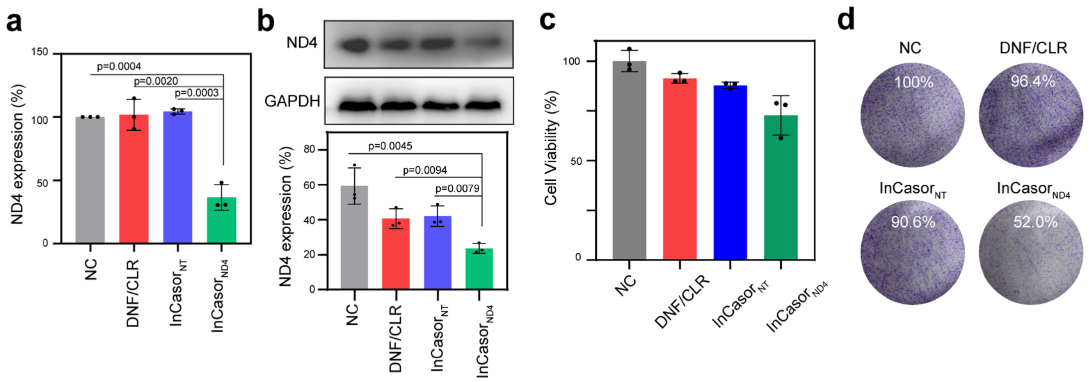

Supplementary Fig. 7. Gene editing efficiency and cytotoxicity of InCasor. a. qPCR analysis of *ND4* gene in the HepG2 cells after incubated with $InCasor_{ND4}$ for 12 h. n=3, data are analyzed by one-way ANOVA with post hoc Tukey's test and shown as mean ± SD. **b.** Western Blot analysis of ND4 expression in the HepG2 cells after incubated with $InCasor_{ND4}$ for 12 h. n=3, data show mean ± SD. n=3, data are analyzed by one-way ANOVA with post hoc Tukey's test and shown as mean ± SD. **c.** Cell viability analysis of HepG2 cells treated with 50 nM DNF/CLR, $InCasor_{NT}$ and $InCasor_{ND4}$. n=3, data show mean ± SD. **d.** Cell cloning experiment of groups corresponding to panel h. The experiments were repeated three times independently. Source data from (a-c) are provided as a Source Data file.

a

Sample ID	InCasor$_{ND4}$	NC	InCasor$_{NT}$
Total Reads Count	1141694958	987035988	862311056
Uniquely mapped	906690699(93.67%)	795437087(93.62%)	1048631488(93.25%)
Reads mapped in proper pairs	900782557(93.06%)	789278765(92.89%)	1043038491(92.76%)
Depth	46.23X	40.52X	53.61X
Coverage	91.22%	91.42%	91.55%
Coverage at least 4X	88.77%	90.24%	91.38%
Coverage at least 10X	80.46%	84.14%	90.67%
Coverage at least 20X	67.08%	68.86%	85.68%

b

Locus	Iden: (%)	nDNA candidate site
Ch.1	95	TTTAGCTCTTCCCCAACCCTTT

c

Sample	Locus	nDNA secquence
NC	Ch.1	TTTAGCTCTTCCCCAACCCTTT
InCasor$_{NT}$	Ch.1	TTTAGCTCTTCCCCAACCCTTT
InCasor$_{ND4}$	Ch.1	TTTAGCTCTTCCCCAACCCTTT

d

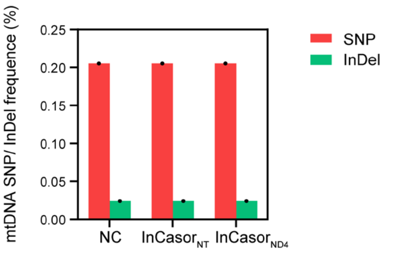

e

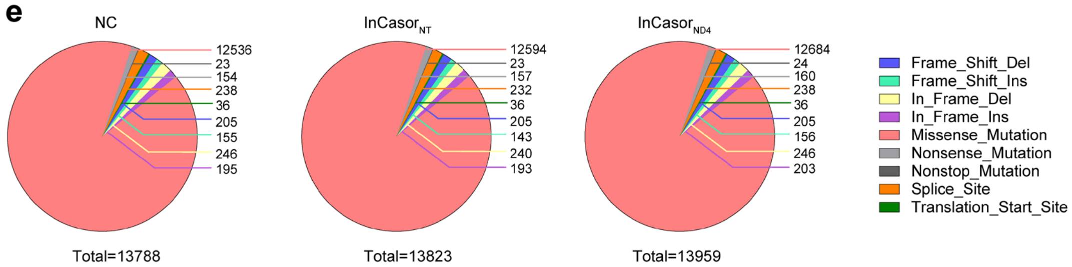

Supplementary Fig. 8. Assessment of mtDNA specific site cleavage and off-target effects of mtDNA and nuclear DNA. a. The overall quality of WGS data. Quality of whole genome sequencing (WGS) data for HepG2 cells treated with 50 nM InCasor$_{NT}$ and InCasor$_{ND4}$. **b.** Sequence of regions in nuclear DNA bearing greatest homology to mtDNA target site. Percentage of sequence identity with the canonical site in mtDNA (Ident.) are shown. The site assessed is the only loci in human nDNA with >75% sequence identity for the InCasor$_{ND4}$ target site in mtDNA. Bases in red indicate divergence from the target sequence. **c.** Analysis of off-target effects of nuclear genome. DNA samples from HepG2 cells treated with 50 nM InCasor$_{NT}$ and InCasor$_{ND4}$ and controls were analyzed. **d.** Percentage of SNP/ InDel frequency of the mtDNA target site from HepG2 cells treated with 50 nM InCasor$_{NT}$ and InCasor$_{ND4}$. **e.** Identification of SNV and indel mutations in a InCasor$_{ND4}$-treated HepG2 cells at the WGS level. Valid sequencing data were aligned to human genome version 19 (hg19).

Notes to Supplementary Fig. 8.

To further examine the effects of InCsaor$_{ND4}$ on the mitochondrial and nuclear genomes, we performed whole-genome sequencing (WGS) on HepG2 cells that were

treated with 50 nM $InCasor_{NT}$ or $InCasor_{ND4}$ (**Supplementary Fig. 8a**). Despite the presence of one region with significant homology to the mtDNA target site in the nuclear genome, no evidence for off-target effects exerted by $InCasor_{ND4}$ could be detected at these sites (**Supplementary Fig. 8 b-c**). In addition, no evidence for non-homologous end joining at the target site in mtDNA could be detected, confirming previous data that $InCasor_{ND4}$ induced double-stand DNA breaks do not result in non-homologous end joining activity (**Supplementary Fig. 8d**). Furthermore, we analyzed the single-nucleotide variants (SNVs) and indels at an average depth of 40.5-, 50.6- and 46.2-fold for NC, $InCasor_{NT}$ and $InCasor_{ND4}$ group, respectively, including frame-shift-del, frame-shift-ins, in-frame-del, in-frame-ins, missense-mutation, nonsense-mutation, nonstop-mutation, splice-site, translation-start-site and intergenic regions. In total, 13959 SNVs and Indels were detected in $InCasor_{ND4}$ group, 13788 SNVs and Indels were detected in NC group and 13823 SNVs and Indels were detected in $InCasor_{NT}$ group (**Supplementary Fig. 8e**). We then filtered SNVs and indels using the 104 Cas-OFFinder predicted off-target sites and the WGS sequence of NC group. None of them were located in the coding region, suggesting no functional off-target sites in $InCasor_{ND4}$ group.

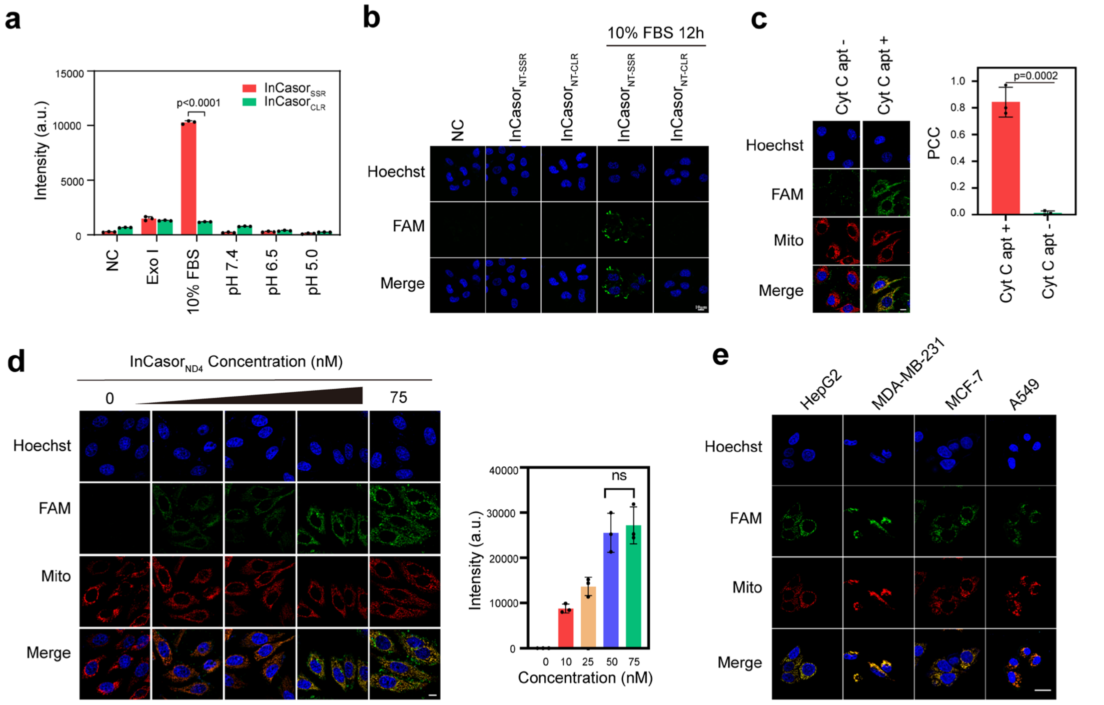

Supplementary Fig. 9. Optimization of InCasor probe for live cell imaging. a. Comparison of stability between InCasor probes with single strand reporter (InCasor$_{SSR}$) and circular reporter (InCasor$_{CLR}$) in different conditions by *in vitro* fluorescence assay. SSR could be cleaved in 10% FBS, releasing high fluorescent signal, while circular reporter maintained high stability in all conditions. n=3, data are analyzed by two-sided Student's t-test and shown as mean ± SD. **b.** Comparison of stability between InCasor$_{ND4\text{-}SSR}$ and InCasor$_{ND4\text{-}CLR}$ for live cell imaging. Scale bar: 25 µm. The experiments were repeated three times independently. **c.** Comparison of InCasor$_{Cyt\ C\ apt+}$ and InCasor$_{Cyt\ C\ apt-}$ for mtDNA imaging. Cyt C aptamer is needed for delivery of Cas12a/crRNA and CLR into mitochondria. PCC (right) of InCasor and mitochondrial were statistically analyzed by Fiji. n=3, data are analyzed by two-sided Student's t-test and shown as mean ± SD. **d.** Confocal imaging of HepG2 cells treated with different concentrations of InCasor$_{ND4}$. Scale bar: 25 µm. Semi-quantitative analysis of fluorescent intensity in each cell is shown on the right. n=3, data show mean ± SD. **e.** Confocal imaging of different cell lines (HepG2, MDA-MB-231, MCF-7, A549 cell lines) treated with 50 nM of the InCasor$_{ND4}$ probe. Scale bar: 10 µm. The experiments were repeated three times independently. Source data from (a, c, and d) are provided as a Source Data file.

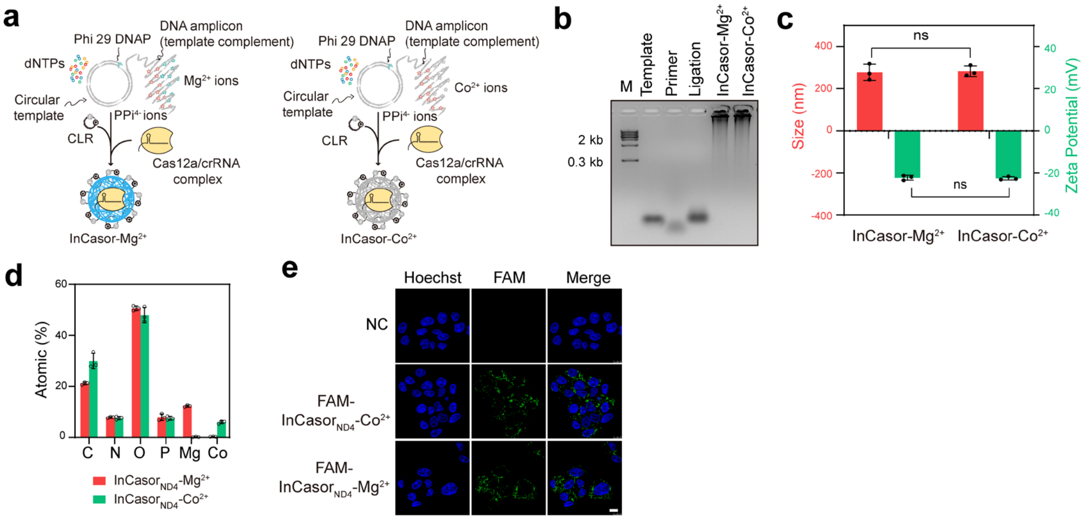

Supplementary Fig. 10. Preparation and characterization of the InCasor$_{ND4}$-Co^{2+} probe. a. Synthesis of InCasor-Co^{2+} probe by replacing Mg^{2+} with Co^{2+} in rolling circle amplification reaction. **b.** Agarose gel electrophoresis analysis of synthesized InCasor-Mg^{2+} and InCasor- Co^{2+} probe: Lane 1: DNA ladder, Lane 2: Template, Lane 3: Primer, Lane 4: Ligation, Lane 5: InCasor-Mg^{2+}, Lane 6: InCasor-Co^{2+}. **c.** Particle sizes and Zeta potential of InCasor-Mg^{2+} and InCasor-Co^{2+} probe. n=3, data are analyzed by two-sided Student's t-test and shown as mean ± SD. **d.** SEM-based EDS characterization of the elemental compositions of the InCasor-Mg^{2+} and InCasor-Co^{2+}. n=3, data show mean ± SD. **e.** Confocal imaging of HepG2 cells incubated with 50 nM FAM-labelled InCasor$_{ND4}$-Mg^{2+} or InCasor-Co^{2+} probe. Scale bar: 10 µm. The experiments were repeated three times independently. Source data from (b-d) are provided as a Source Data file.

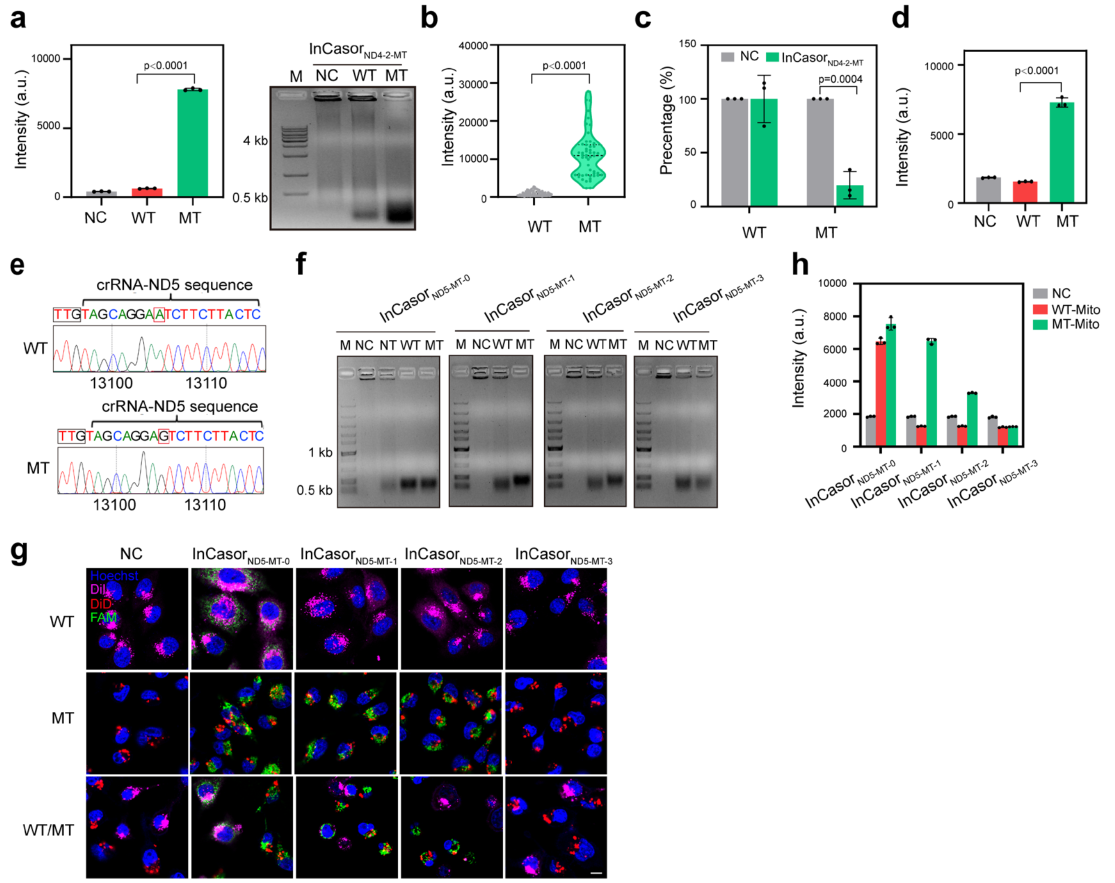

Supplementary Fig. 11. Design InCasor for identifying mutations in mtDNA. a. Fluorescence analysis and agarose gel electrophoresis analysis of InCasor$_{\text{ND4-2-MT}}$ probe (designed for targeting 12084C>T mutation in mtDNA) incubated with wild-type (WT) or mutant (MT) dsDNA. n=3, data show mean ± SD. **b.** Semi-quantitative statistics of fluorescence intensity of HepG2 cells (WT) and MDA-MB-231 cells (MT mtDNA) treated by InCasor$_{\text{ND4-2-MT}}$. Scale bar: 10 μm. n=50. **c.** qPCR analysis of *ND4* gene in the HepG2 cells (WT) and MDA-MB-231 cells (MT) incubated with InCasor$_{\text{ND4-2-MT}}$ probe for 12 h. n=3, data show mean ± SD. **d.** Fluorescence analysis of detecting mutant mtDNA (12084C>T) in mitochondria extracted from MDA-MB-231 cells using InCasor$_{\text{ND4-2-MT}}$. WT-Mito were extracted from HepG2 cells and used as control. n=3, data show mean ± SD. **e.** Sequencing result of mtDNA at position 13105 in HepG2 and MDA-MB-231 cells. MDA-MB-231 cells holds 13105A>G mutation in *ND5* gene. **f.** Agarose gel electrophoresis analysis of InCasor probe with different crRNA after incubation with WT or MT dsDNA. **g.** Imaging of 13105A>G point mutation in mtDNA in HepG2 and MDA-MB-231 cells. Cell nucleus are shown in blue, HepG2 cells were labelled with DiI (purple), MDA-MB-231 cells were labelled with DiD (red) and the InCasor probe would generate fluorescent signal when binding target mtDNA (green). Scale bar: 10 μm. Semi-quantitative statistics are performed on the three types of fluorescence intensities (DiI, DiD and FAM) of each cell in the co-culture group. (n=50). **h.** Detecting mutant mtDNA (13105A>G) in mitochondria extracted from MDA-MB-231 cells using InCasor with different crRNA. WT-Mito were extracted from HepG2 cells and used as control. n=3, data show mean ± SD. For panel a-d, data are analyzed

by two-sided Student's t-tests. Source data from (a-d, and f-h) are provided as a Source Data file.

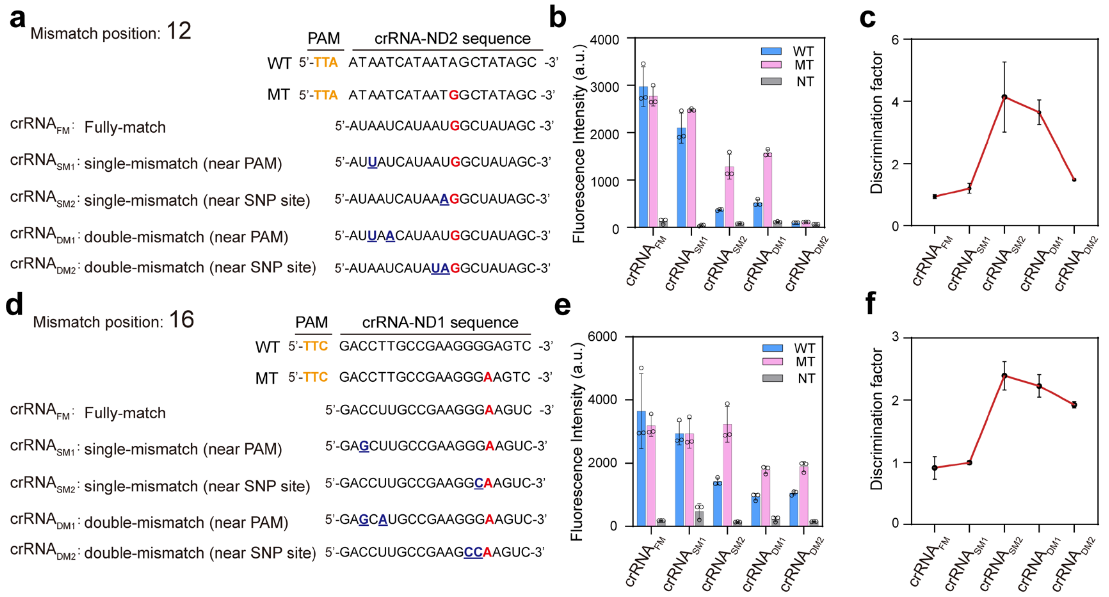

Supplementary Fig. 12. Engineering crRNA for recognition of mutations in mtDNA that are distant from the PAM. a. Sequences of crRNA$_{FM}$ (fully matched with the 4769A>G mutant *ND2* gene) and engineered crRNA$_s$ (with different numbers of nucleotide mismatches at different position). Each SNV is indicated by a bold red letter. Each mismatched position is indicated by an underlined bold blue letter. **b.** An *in vitro* fluorescence assay was used to estimate the ability of different InCasor probes to detect the 4769A>G mutation in the *ND2* gene. n=3, data show mean ± SD. **c.** The discrimination factors of the InCasor probe with different crRNAs toward WT and MT mtDNA at 4769A>G. n=3, data show mean ± SD. **d.** Sequences of crRNA$_{FM}$ (fully matched with the 3916G>A mutant *ND1* gene) and engineered crRNA$_s$ (with different numbers of nucleotide mismatches at different position). Each SNV is indicated by a bold red letter. Each mismatched position is indicated by an underlined bold blue letter. **e.** An *in vitro* fluorescence assay was used to estimate the ability of different InCasor probes to detect the 3916G>A mutation in the *ND1* gene. n=3, data show mean ± SD. **f.** The discrimination factors of the InCasor probe with different crRNAs toward WT and MT mtDNA at 3916G>A. n=3, data show mean ± SD. The sequences of all crRNAs used are listed in Supplementary Table 1. Source data from (b, c, e, and f) are provided as a Source Data file.

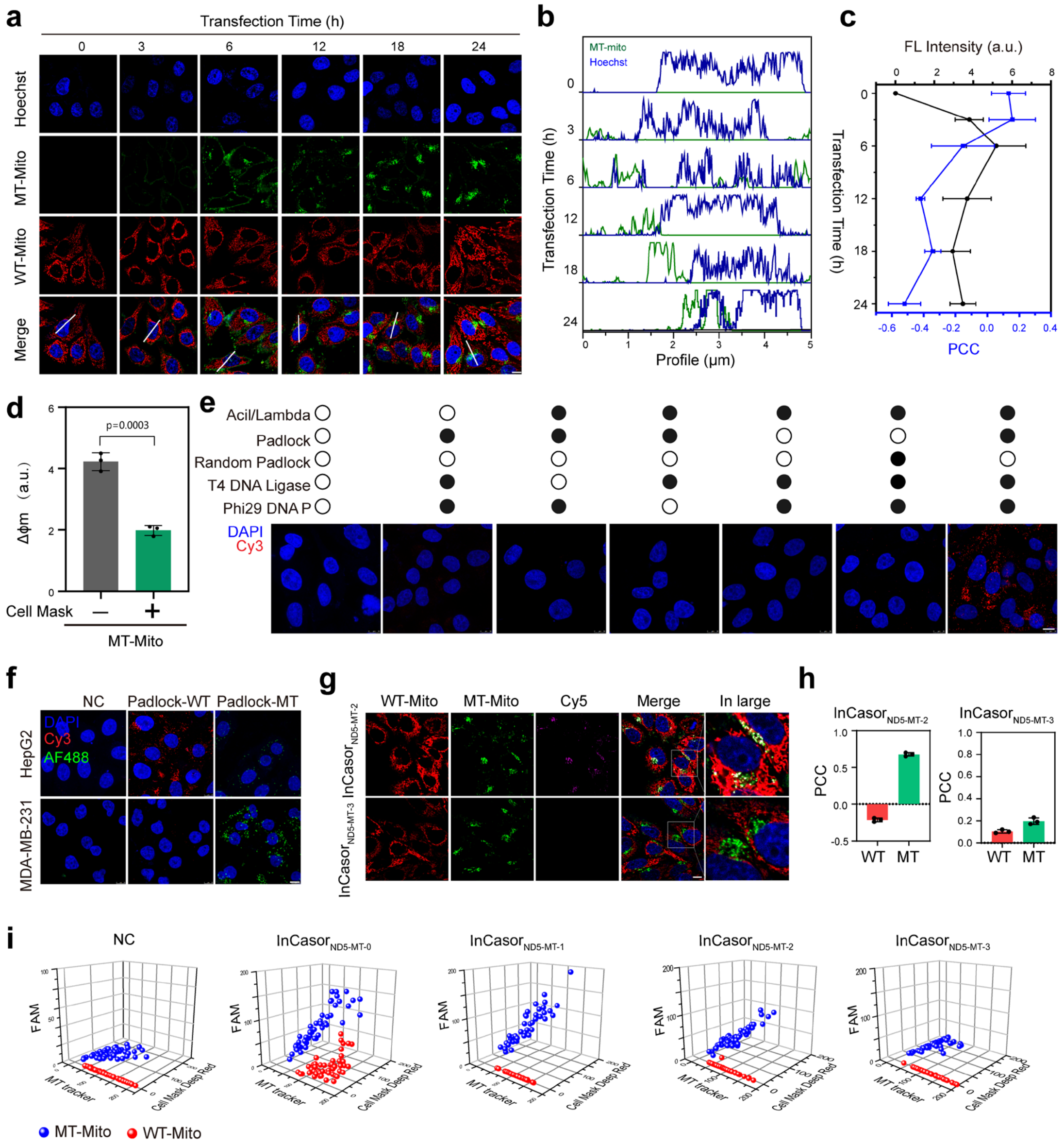

Supplementary Fig. 13. Construction and validation of cells with heteroplasmic mtDNA. a. Confocal imaging of HepG2 cells incubated with 1.0 μg MT-Mito for different times. Cell nuclei are stained blue by Hoechst 33342, WT-Mito were labeled with MitoTracker (red), and MT-Mito were pre-stained with CellMask (green); Scale bar: 25 μm. **b.** The spatial co-localization of cell nuclei(blue) and MT-Mito(green) were statically analyzed by Fiji. **c.** Semi-quantitatively analyze (black) the changes of MT-mito fluorescence intensity and the PCC analyze (blue) the changes of three kinds of fluorescence signal corresponding to panel a with transfection time by Fiji. n=3, data show mean ± SD. **d.** Mitochondrial membrane potential (Δϕm) measurements of isolated MT-Mito stained with/without membrane dye. Isolated MT-Mito were treated with Cell Mask Green for 15min and the free dye was removed by centrifugation. Data are analyzed by two-sided Student's t-test and shown as mean ± SD. (n = 3). **e.** Assess the feasibility of in situ RCA to image mitochondrial DNA in cultured cells. Nuclei were stained with DAPI (blue) and Cy3-labeled padlock probes hybridized and ligated to the target sequence were visualized by immunohistochemistry (red). Scale bar: 10

μm. **f.** Genotyping of the HepG2 and MDA-MB-231 cell lines, homoplasmic for the two different genotypes. Nuclei were stained with DAPI (blue), Cy3-labeled padlock probes on wild-type mitochondrial DNA (red) and AF488-labeled padlock probes on mutant mitochondrial DNA (green). Scale bar: 10 μm. **i.** In situ genotyping of the heterogeneous cells. Nuclei were labelled by DAPI (blue), AF488-labeled padlock probes on wild-type mitochondrial DNA (red) and Cy3-labeled padlock probes on mutant mitochondrial DNA (green). Scale bar: 10 μm. **g.** Analyzing heteroplasmic mtDNA mutations in live cybrid cells using InCasor with $crRNA_2$ or $crRNA_3$. Scale bar: 2.5 μm. Cell nucleus are shown in blue, WT-Mito were labelled by Mito-Tracker (red), InCasor probe would generate fluorescent signal (green) when binding targeted mtDNA. Scale bar: 10 μm. **h.** PCC of purple signal with green or red fluorescence in panel g were statically analyzed. n=3, data show mean ± SD. **i.** Semi-quantitative statistics are performed on the three types of fluorescence intensities (MT-Tracker, cell mask deep red and FAM) of each mitochondrial in the live cybrid cells. (n=50). Source data from (b, c, d, h, and i) are provided as a Source Data file.

Notes to Supplementary Fig. 13

We created cells with heteroplasmic mtDNA, i.e., containing both mitochondria with wild-type mtDNA (WT-Mito) and mitochondria with mutant mtDNA (MT-Mito), by isolating mitochondria from MDA-MB-231 cells and transfecting them into HepG2 cells. Before that, we first examined mitochondrial DNA heterogeneity in HepG2 cells and MDA-MB-231 cells separately by pyrosequencing (**Fig. 5a**). WT-Mito were labeled with MitoTracker (red), and MT-Mito were pre-stained with CellMask (green). During 24 hours of internalization, labeled MT-Mito first bound to the cell surface (3-6 hours), then entered the cytoplasm (12 hours), and finally localized near the nucleus (18-24 hours) (**Supplementary Fig. 13a**). The spatial co-localization of nuclei (blue) and MT-Mito (green) from static analysis in Fiji also indicated that the distribution, or motility, of labeled MT-Mito in HepG2 cells was nucleotropic (**Supplementary Fig. 13b**). Meanwhile, no co-localization of MT-Mito and WT-Mito was observed, and the PCC between them was always less than 0 (**Supplementary Fig. 13c**). We speculated that MT-Mito is compromised during extraction and fluorescent labeling to tend to the vicinity of the nucleus where mitophagy occurs (**Supplementary Fig. 13d**).

To further determine mitochondrial DNA heterogeneity and its stability in heterogeneous cells, we investigated its heterogeneity using *in situ* RCA and pyrosequencing, respectively. We used a pair of padlock probes specific for the two

sequence variants to genotype the mitochondrial 13105A>G point mutation in situ in heterogeneous cells. RCA products were detected by hybridization of two fluorescent oligonucleotide probes with sequences identical to distinct tag sequences in the two variant-specific padlock probes. As shown in **Supplementary Fig. 13 e-f**, the strong discrete signal shows the distribution of the two mitochondrial genome variants in the heterogeneous cells.

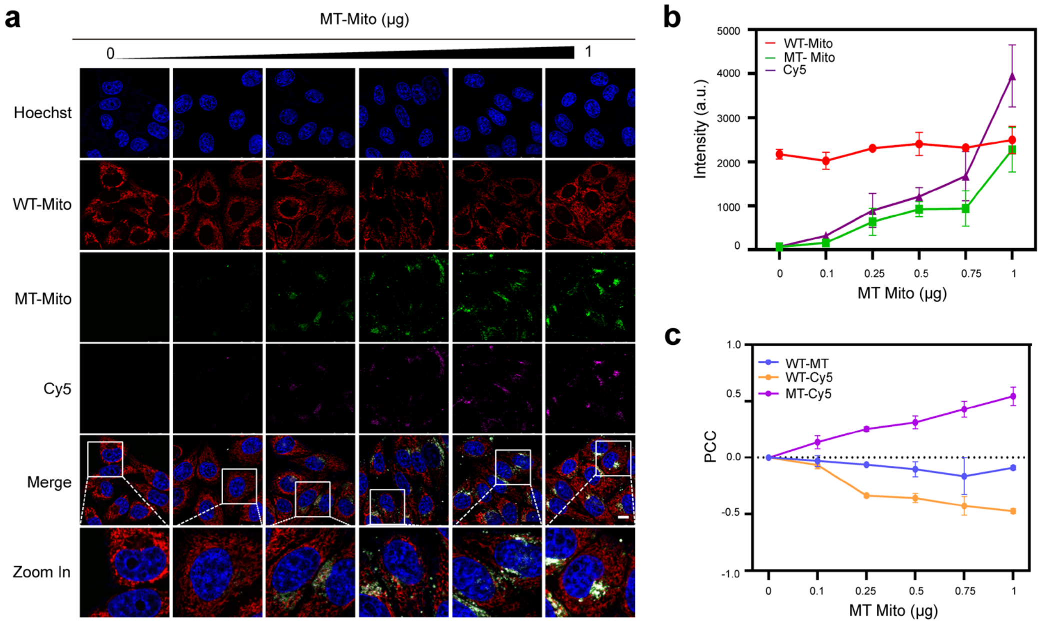

Supplementary Fig. 14. Imaging analysis of the heterogeneity of the 13105A>G mutation in the *ND5* gene in mtDNA in hybrid cells using InCasor$_{\mathrm{ND5\text{-}MT\text{-}1}}$. a. Representative images of HepG2 cells transfected with different amounts of MT-Mito and then treated with InCasor$_{\mathrm{ND5\text{-}MT\text{-}1}}$. Cell nuclei are stained blue by Hoechst 33342, WT-Mito were labeled with MitoTracker (red), and MT-Mito were pre-stained with CellMask (green); the fluorescence generated by InCasor$_{\mathrm{ND5\text{-}MT\text{-}1}}$ (Cy5) appears purple. Scale bar: 25 μm. **b.** Semi-quantitatively analyzed the changes of three kinds of fluorescence intensity corresponding to Fig. 5f with mitochondrial concentration by Fiji. n=3, data show mean ± SD. **c.** PCC analyze the changes of three kinds of fluorescence signal corresponding to Fig. 5f with mitochondrial concentration by Fiji. n=3, data show mean ± SD. Source data from (b, c) are provided as a Source Data file.

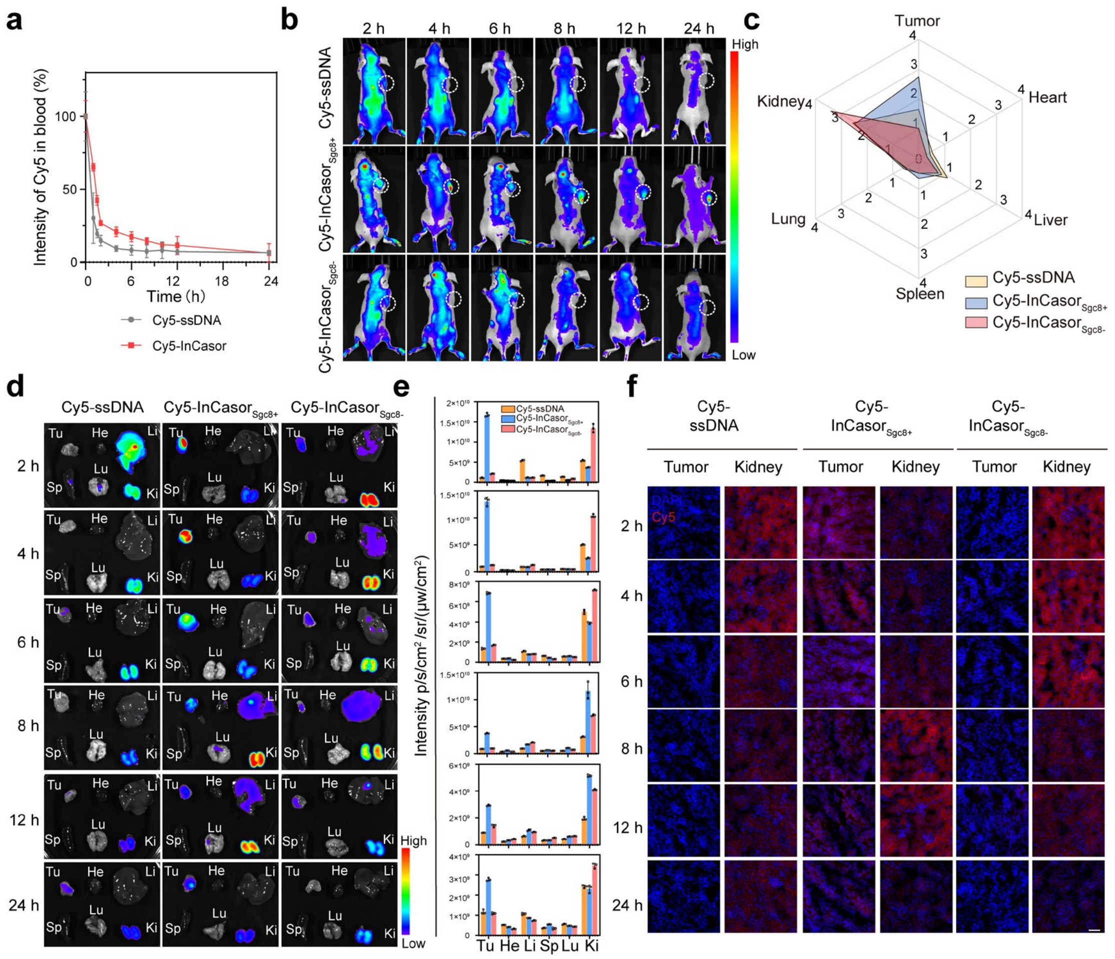

Supplementary Fig. 15. Analysis of the *in vivo* biodistribution of InCasor. a. Blood circulation curve of ssDNA and InCasor by measuring the fluorescence intensity of Cy5 in blood at different time points post-injection (n=3, data show mean ± SD.). **b.** *In vivo* biodistribution of Cy5-labelled ssDNA, InCasor$_{Sgc8+}$, and InCasor$_{Sgc8-}$ in HepG2 tumor-bearing mice over 24 h. **c.** Semi-quantitative analysis of fluorescence intensity of harvested tumor and major organs at 24 h from panel b. n=3, data show mean ± SD. **d.** *Ex vivo* imaging of major organs harvested from HepG2 tumor-bearing mice at 2, 4, 6, 8, 12, and 24 h post injection of Cy5-ssDNA, Cy5- InCasor$_{Sgc8+}$, and Cy5- InCasor$_{Sgc8-}$. **e.** Semi-quantitative analysis of d. n=3, data show mean ± SD. **f.** Representative images of the tumor, liver, and kidney tissue section in d. Scale bar: 50 μm. Source data from (a, c, and e) are provided as a Source Data file.

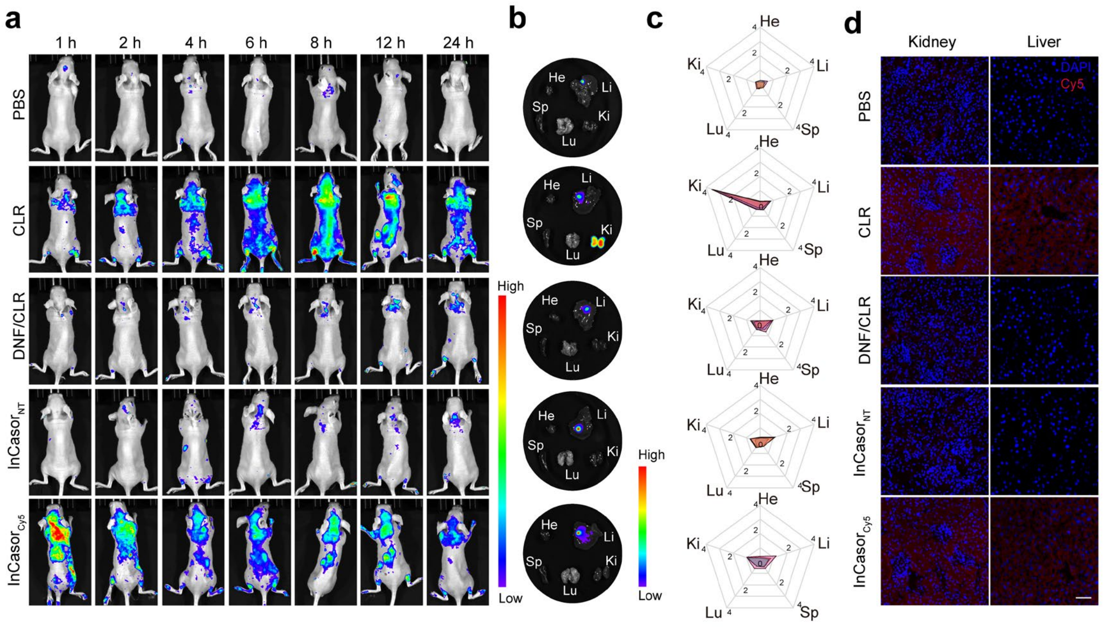

Supplementary Fig. 16. Analysis of the *in vivo* stability of InCasor. a. *In vivo* fluorescence signals of CLR, DNF/CLR, InCasor$_{NT}$, and InCasor$_{Cy5}$ in HepG2 tumor-bearing mice over 24 h. **b.** Fluorescence imaging of harvested tumor and major organs (heart; liver; spleen; lung; and kidney) after 24 h post tail-vein injection of different probe. **c.** Semi-quantitative analysis of b. n=3, data show mean ± SD. **d.** Representative images of the tumor, liver, and kidney tissue section in d. Scale bar: 50 μm. Source data from c are provided as a Source Data file.

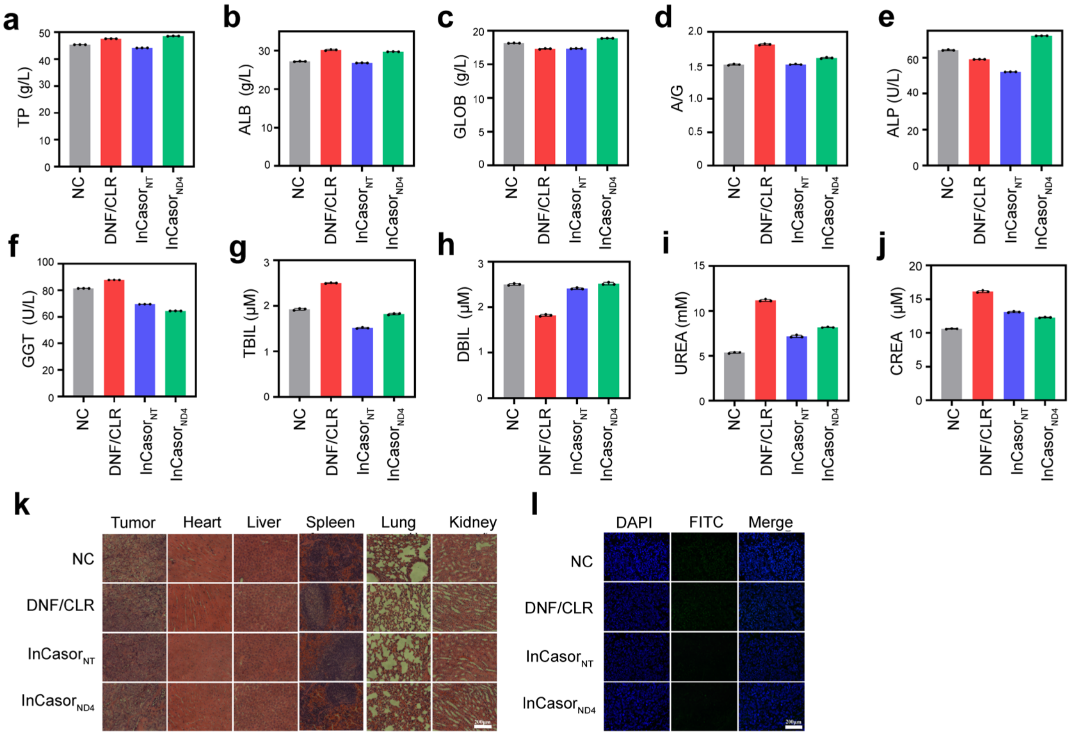

Supplementary Fig. 17. Analysis of *in vivo* safety of InCasor. a-j. Blood biochemistry and hematology analysis of the mice after treatment with InCasor for 8 hours. n=3, data show mean ± SD. **k-l.** H&E staining (k) of major organ sections and TUNEL staining (l) of tumor sections after indicated treatments. Scale bar: 200 μm. Source data from (a-j) are provided as a Source Data file.

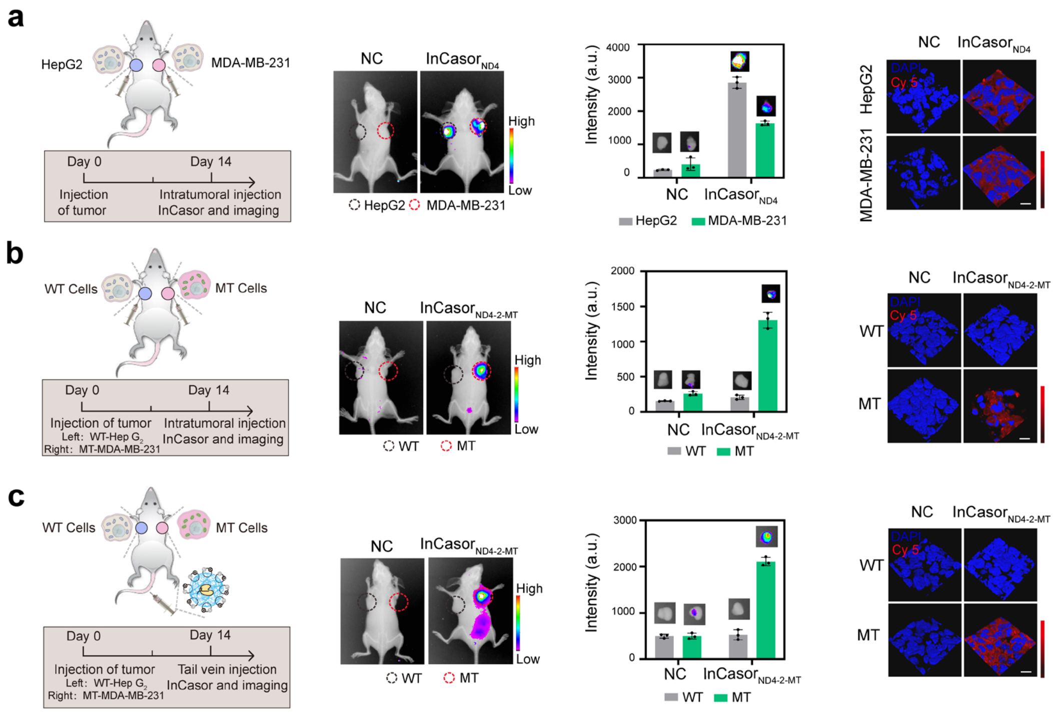

Supplementary Fig. 18. Visualizing mtDNA mutation (12084C>T, SNV in PAM) *in vivo* using InCasor. a. Schematic of a dual tumor mice model to test the capability of InCasor to image targeted mtDNA in live mice. Construction process of dual tumor mouse model, administration method and imaging time are indicated in gray box. *In vivo* imaging, fluorescence imaging of tumor organ and tumor tissue slices all show that InCasor is able to detect specific mtDNA target in HepG2 and MDA-MB-231 tumors. n=3, data show mean ± SD. Scale bar: 25 μm. **b.** Schematic of dual tumor mice model to test the ability of InCasor to image SNV in mtDNA (12084C>T) *in vivo* by intratumorally injection. *In vivo* imaging, fluorescence imaging of tumor organ and tumor tissue slices all show that InCasor is able to precisely image SNV in mtDNA (12084C>T) *in vivo*. n=3, data show mean ± SD. Scale bar: 25 μm. **c.** Schematic of dual tumor mice model to test the ability of InCasor to image SNV in mtDNA (12084C>T) *in vivo* by tail vein injection. *In vivo* imaging, fluorescence imaging of tumor organ and tumor tissue slices all show that InCasor is able to image SNV in mtDNA (12084C>T) *in vivo* by tail-vein injection of InCasor probe. n=3, data show mean ± SD. Scale bar: 25 μm. Source data from (a, b, and c) are provided as a Source Data file.

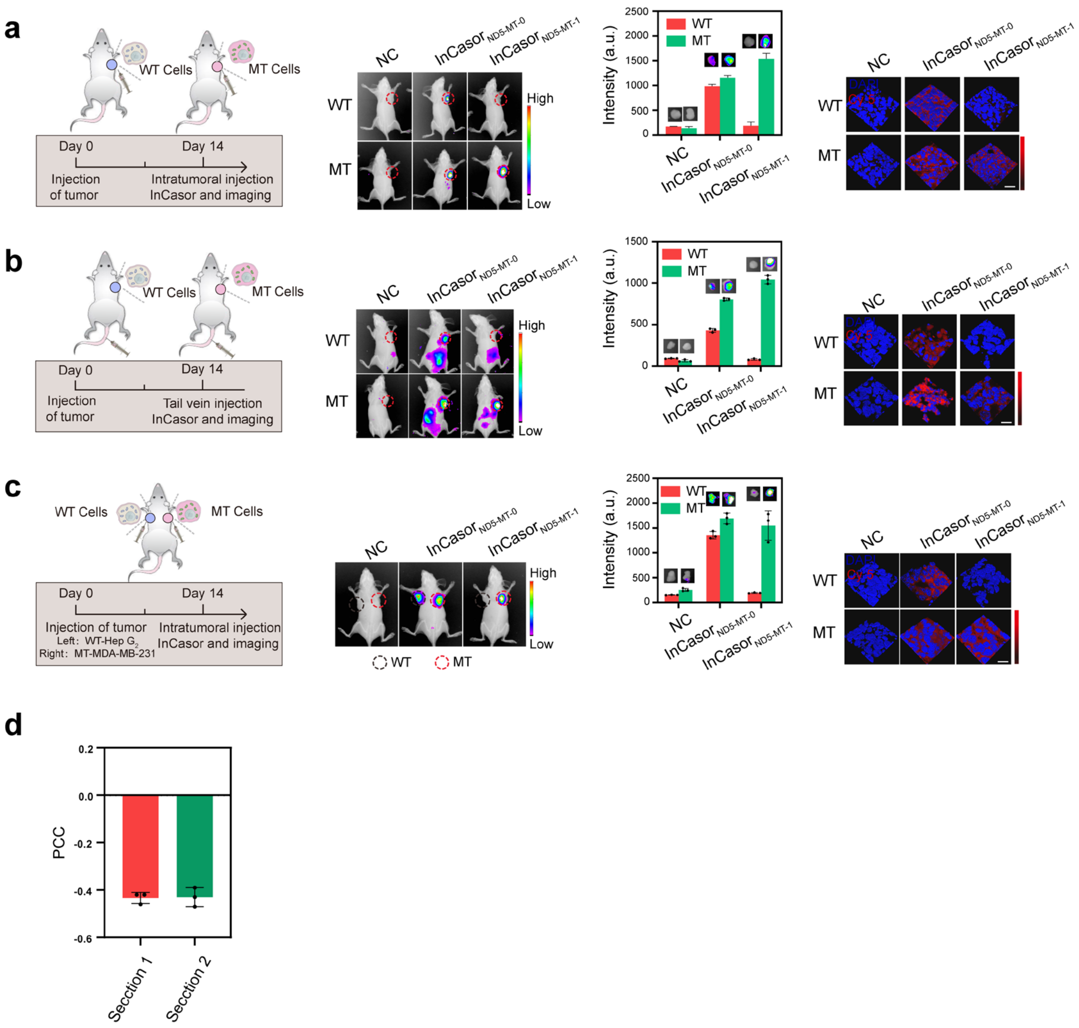

Supplementary Fig. 19. Visualizing mtDNA mutation (13105A>G, SNV in near-PAM) *in vivo* using InCasor. a. Schematic of tumor-bearing mice model to test the ability of InCasor with different crRNA ($crRNA_0$ and $crRNA_1$) to image 13105A>G SNV in mtDNA *in vivo* by intratumorally injection. Construction process of tumor-bearing mouse model, administration method and imaging time are indicated in gray box. *In vivo* imaging, fluorescence imaging of tumor organ and tumor tissue slices indicate that InCasor with engineered crRNA ($crRNA_1$) show higher specificity for imaging 13105A>G SNV in mtDNA *in vivo*. Scale bar: 25 µm. n=3, data show mean ± SD. **b.** Schematic of tumor-bearing mice model to test the ability of InCasor with different crRNA ($crRNA_0$ and $crRNA_1$) to image 13105A>G SNV in mtDNA *in vivo* by tail vein injection. *In vivo* imaging, fluorescence imaging of tumor organ and tumor tissue slices indicate that InCasor with engineered crRNA ($crRNA_1$) is able to specifically image SNV in near-PAM (13105A>G) *in vivo* via tail-vein injection. Scale bar: 25 µm. n=3, data show mean ± SD. **c.** Schematic of dual tumor mice model to test the ability of InCasor with different crRNA ($crRNA_0$ and $crRNA_1$) to image 13105A>G SNV in mtDNA *in vivo* by intratumorally injection. *In vivo* imaging, fluorescence imaging of tumor organ and tumor tissue slices all indicate that InCasor with engineered crRNA ($crRNA_1$) is able to specifically image SNV in near-PAM (13105A>G) *in vivo*

via tail-vein injection. Scale bar: 25 μm. n=3, data show mean ± SD. The length and width of the slices are both 2000 μm. **d.** PCC of InCasor and Arginase-1 in Fig. 6h were statically analyzed by Fiji. Source data from (a, b, c, and d) are provided as a Source Data file.

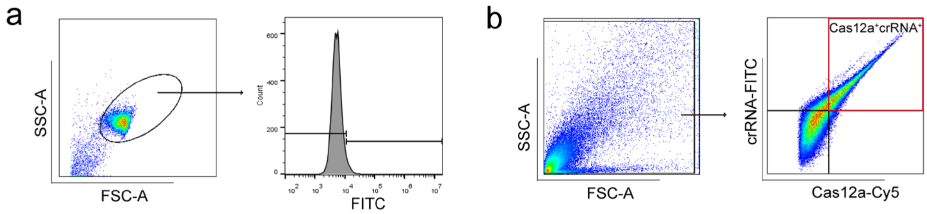

Supplementary Fig. 20. Gating strategy for this work. a. Gating strategies to analyze HepG2 cells. **b.** Gating strategies to analyze mitochondria.

Supplementary References

1 Hu, M. *et al.* Single-Step, Salt-Aging-Free, and Thiol-Free Freezing Construction of AuNP-Based Bioprobes for Advancing CRISPR-Based Diagnostics. *J Am Chem Soc* **142**, 7506-7513, (2020).

2 Larsson, C. *et al.* In situ genotyping individual DNA molecules by target-primed rolling-circle amplification of padlock probes. *Nat Methods* **1**, 227-232, (2004).

3 Deng, R. *et al.* DNA-Sequence-Encoded Rolling Circle Amplicon for Single-Cell RNA Imaging. *Chem* **4**, 1373-1386, (2018).

4 Sun, W. *et al.* Cocoon-Like Self-Degradable DNA Nanoclew for Anticancer Drug Delivery. *Journal of the American Chemical Society* **136**, 14722-14725, (2014).
