# Supplementary material for: In vivo imaging of mitochondrial DNA mutations using an integrated nano Cas12a sensor
Source: Nat Commun. 2023 Nov 24;14:7722. doi: 10.1038/s41467-023-43552-0 (PMC10673915; doi:10.1038/s41467-023-43552-0)
Supplement: Supplementary file 3 — Reporting Summary [file 41467_2023_43552_MOESM3_ESM.pdf]

nature portfolio

Corresponding author(s): Kaixiang Zhang

Last updated by author(s): Oct 7, 2023

Reporting Summary

Nature Portfolio wishes to improve the reproducibility of the work that we publish. This form provides structure for consistency and transparency in reporting. For further information on Nature Portfolio policies, see our Editorial Policies and the Editorial Policy Checklist.

Statistics

For all statistical analyses, confirm that the following items are present in the figure legend, table legend, main text, or Methods section.

n/a	Confirmed	
☐	☒	The exact sample size (*n*) for each experimental group/condition, given as a discrete number and unit of measurement
☐	☒	A statement on whether measurements were taken from distinct samples or whether the same sample was measured repeatedly
☐	☒	The statistical test(s) used AND whether they are one- or two-sided<br>*Only common tests should be described solely by name; describe more complex techniques in the Methods section.*
☒	☐	A description of all covariates tested
☒	☐	A description of any assumptions or corrections, such as tests of normality and adjustment for multiple comparisons
☐	☒	A full description of the statistical parameters including central tendency (e.g. means) or other basic estimates (e.g. regression coefficient) AND variation (e.g. standard deviation) or associated estimates of uncertainty (e.g. confidence intervals)
☐	☒	For null hypothesis testing, the test statistic (e.g. *F*, *t*, *r*) with confidence intervals, effect sizes, degrees of freedom and *P* value noted<br>*Give P values as exact values whenever suitable.*
☒	☐	For Bayesian analysis, information on the choice of priors and Markov chain Monte Carlo settings
☒	☐	For hierarchical and complex designs, identification of the appropriate level for tests and full reporting of outcomes
☐	☒	Estimates of effect sizes (e.g. Cohen's *d*, Pearson's *r*), indicating how they were calculated

Our web collection on statistics for biologists contains articles on many of the points above.

Software and code

Policy information about availability of computer code

Data collection	LEICA TCS SP8 STED and LAS AF Lite software (NIS ElementsAR ver. 5.02.01). FlowJo software (version 10.4). Bruker MI SE (version 721)
Data analysis	ImageJ/Fiji (version 2.0.0-rc-69/1.52p) were used to align, normalize, contrast, overlay, and measure images as described in the Methods section.GraphPad Prizm (version 8.2) was used to generate plots. Microsoft Excel (version 16.16.7) was used to generate tables. Adobe Illustrator (version 22.0.1) was used to assemble figures. LAS AF Lite software(NIS ElementsAR ver. 5.02.01.) was used to process images and 3D reconstruction. Origin were used to generate Three- Dimensional Graph. FlowJo software (version 10.4). Bruker MI SE (version 721).

For manuscripts utilizing custom algorithms or software that are central to the research but not yet described in published literature, software must be made available to editors and reviewers. We strongly encourage code deposition in a community repository (e.g. GitHub). See the Nature Portfolio guidelines for submitting code & software for further information.

Data

Policy information about availability of data

All manuscripts must include a data availability statement. This statement should provide the following information, where applicable:

- Accession codes, unique identifiers, or web links for publicly available datasets
- A description of any restrictions on data availability
- For clinical datasets or third party data, please ensure that the statement adheres to our policy

All the data of this study are available within the article and Supplementary Information files or from the corresponding author on request Source data are provided

with this paper. All next-generation sequencing data generated in this study are accessible from the BioProject database using the accessions PRJNA807948(https://dataview.ncbi.nlm.nih.gov/object/PRJNA807948), PRJNA807889(https://dataview.ncbi.nlm.nih.gov/object/PRJNA807889), and PRJNA807862 (https://dataview.ncbi.nlm.nih.gov/object/PRJNA807862). Source data are provided with this paper.

Research involving human participants, their data, or biological material

Policy information about studies with human participants or human data. See also policy information about sex, gender (identity/presentation), and sexual orientation and race, ethnicity and racism.

Reporting on sex and gender	Not applicable.
Reporting on race, ethnicity, or other socially relevant groupings	Not applicable.
Population characteristics	Not applicable.
Recruitment	Not applicable.
Ethics oversight	Not applicable.

Note that full information on the approval of the study protocol must also be provided in the manuscript.

Field-specific reporting

Please select the one below that is the best fit for your research. If you are not sure, read the appropriate sections before making your selection.

☒ Life sciences ☐ Behavioural & social sciences ☐ Ecological, evolutionary & environmental sciences

For a reference copy of the document with all sections, see nature.com/documents/nr-reporting-summary-flat.pdf

Life sciences study design

All studies must disclose on these points even when the disclosure is negative.

Sample size	No sample size calculations were performed, as our goal was to obtain as much experimental data as possible during technical optimisation, taking into account the limitations of the experiment. For all analytical experiments, at least three technical replicates were performed to confirm reproducibility. Due to the low variability observed between samples, we considered this to be sufficient.
Data exclusions	No data was excluded.
Replication	Biological experiments or replicate experiments in triplicate were done with different sample aliquots, with intervals ranging from a few weeks to several months. All experiments were repeated at least once and all attempts were successful.
Randomization	The areas used for imaging were selected randomly . All cell samples that passed quality filters were used for analysis. Mice were grouped in completely randomized groups according to the following steps: mice were numbered; different random numbers were assigned to all mice; random numbers were arranged in excel, and animals were numbered in ascending order of the random numbers; 5 mice were randomly assigned to each experimental group in order.
Blinding	Associated evaluations, such as histological changes, were performed by two researchers blinded to the evaluation. In addition to histological evaluations, researchers were blinded to group assignment during data collection and analysis.

Reporting for specific materials, systems and methods

We require information from authors about some types of materials, experimental systems and methods used in many studies. Here, indicate whether each material, system or method listed is relevant to your study. If you are not sure if a list item applies to your research, read the appropriate section before selecting a response.

Materials & experimental systems

n/a	Involved in the study	
☐	☒	Antibodies
☐	☒	Eukaryotic cell lines
☒	☐	Palaeontology and archaeology
☐	☒	Animals and other organisms
☒	☐	Clinical data
☒	☐	Dual use research of concern
☒	☐	Plants

Methods

n/a	Involved in the study	
☒	☐	ChIP-seq
☐	☒	Flow cytometry
☒	☐	MRI-based neuroimaging

Antibodies

Antibodies used	Anti-ND4 (Abcam: ab219822) used at 1:50.<br>Anti-GAPDH (Abcam: ab128915) used at 1:1000<br>Anti-Arginase-1 (CST: 93668T) used at 1:50<br>Anti-LbCas12a (CST: 41874S) used at 1:1000<br>Anti-MFN2 (Proteintech: 12186) used at 1:5000<br>Anti-HSP 60 (Santa Cruz Biotechnology: sc-57840) used at 1:500
Validation	Anti-ND4 has been validated by Abcam to be specific in MCF-7 and Hep G2 cell lines using western blotting (https://www.abcam.cn/products/primary-antibodies/nd4-antibody-9e4-2d8-n-terminal-ab219822.html).<br>Anti-GAPDH has been validated by Abcam to produce positive signal in whole cell lysates from MCF-7 and Hep G2 cell lines using western blotting (https://www.abcam.cn/gapdh-antibody-epr6256-loading-control-ab128915.html).<br>Anti-Arginase-1 has been validated by Cellsignal to produce positive immunofluorescence signal in Mouse liver tissue section (https://www.cellsignal.cn/products/primary-antibodies/arginase-1-d4e3m-xp-rabbit-mab/93668?_=1622625850954&Ntt=Arginase&tahead=true).<br>Anti-LbCas12a has been validated by Cellsignal to produce positive signal in whole cell lysates or mito lysates from Hep G2 cell lines using western blotting (https://www.cellsignal.cn/products/primary-antibodies/lbcpf1-strain-nd2006-e8b1w-rabbit-mab/41874?site-search-type=Products&N=4294956287&Ntt=41874+lbcpf1+%28strain+nd2006%29+%28e8b1w%29+rabbit+mab&fromPage=plp&_requestid=1542448).<br>Anti-MFN2 has been validated by Proteintech to produce positive signal in mito lysates from Hep G2 cell lines using western blotting (https://www.ptgcn.com/products/MFN2-Antibody-12186-1-AP.htm)<br>Anti-HSP 60 has been validated by Santa Cruz Biotechnology to produce positive signal in mito lysates from Hep G2 cell lines using western blotting (https://www.scbt.com/p/hsp-60-antibody-a57-b9?requestFrom=search)

Eukaryotic cell lines

Policy information about cell lines and Sex and Gender in Research

Cell line source(s)	ATCC HepG2 (HB-8065), ATCC MDA-MB-231 (HTB-26), ATCC MCF-7 (HTB-22), ATCC A549 (CCL-185)
Authentication	Cells were authenticated by the supplier by STR analysis.
Mycoplasma contamination	Cell lines were not tested for Mycoplasma contamination
Commonly misidentified lines (See ICLAC register)	No commonly misidentified cell lines were used.

Animals and other research organisms

Policy information about studies involving animals; ARRIVE guidelines recommended for reporting animal research, and Sex and Gender in Research

Laboratory animals	Female BALB/c nude mice (5-weeks-old) were bought from Hunan SJA Laboratory Animal Co., Ltd and feed at the condition of 25°C and 55% of humidity with 12 h light/dark cycle in Experimental Animal Center of Zhengzhou University. The license number is SCXK (xiang) 2019-0004.
Wild animals	The study did not involve wild animals.
Reporting on sex	This study is generally applicable to different sexes.
Field-collected samples	The study did not involve samples collected form the filed.
Ethics oversight	Animal experiment protocols were conducted in accordance with the guidelines of the regional Animal Experimentation Ethics Committee and Zhengzhou University. The animal laboratory's accreditation number is 110 322 211 102 955 054.

Note that full information on the approval of the study protocol must also be provided in the manuscript.

Flow Cytometry

Plots

Confirm that:

☒ The axis labels state the marker and fluorochrome used (e.g. CD4-FITC).

☒ The axis scales are clearly visible. Include numbers along axes only for bottom left plot of group (a 'group' is an analysis of identical markers).

☐ All plots are contour plots with outliers or pseudocolor plots.

☒ A numerical value for number of cells or percentage (with statistics) is provided.

Methodology

Sample preparation	Hep G2 cells, MDA-MB-231 cells, and MCF-7 cells were incabuated with 50nM InCasor in cell culture media for 6hr.
Instrument	BD FACSVerse™ Accuri C6 system
Software	Aquisition: BD FACSDiva software. Analysis: Flow Jo Software.
Cell population abundance	10000
Gating strategy	Gating strategy for all experiments is shown in Supplementary Fig. 20.

☒ Tick this box to confirm that a figure exemplifying the gating strategy is provided in the Supplementary Information.
